# Transplanting Supersites of HIV-1 Vulnerability

**DOI:** 10.1371/journal.pone.0099881

**Published:** 2014-07-03

**Authors:** Tongqing Zhou, Jiang Zhu, Yongping Yang, Jason Gorman, Gilad Ofek, Sanjay Srivatsan, Aliaksandr Druz, Christopher R. Lees, Gabriel Lu, Cinque Soto, Jonathan Stuckey, Dennis R. Burton, Wayne C. Koff, Mark Connors, Peter D. Kwon

**Affiliations:** 1 Vaccine Research Center, National Institute of Allergy and Infectious Diseases, National Institutes of Health, Bethesda, Maryland, United States of America; 2 Department of Immunology and Microbial Science and IAVI Neutralizing Antibody Center, and Center for HIV/AIDS Vaccine Immunology and Immunogen Design, The Scripps Research Institute, La Jolla, California, United States of America; 3 Ragon Institute of MGH, MIT and Harvard, Cambridge, Massachusetts, United States of America; 4 International AIDS Vaccine Initiative (IAVI), New York, New York, United States of America; 5 HIV-Specific Immunity Section, National Institute of Allergy and Infectious Diseases, National Institutes of Health, Bethesda, Maryland, United States of America; Mayo Clinic, United States of America

## Abstract

One strategy for isolating or eliciting antibodies against a specific target region on the envelope glycoprotein trimer (Env) of the human immunodeficiency virus type 1 (HIV-1) involves the creation of site transplants, which present the target region on a heterologous protein scaffold with preserved antibody-binding properties. If the target region is a supersite of HIV-1 vulnerability, recognized by a collection of broadly neutralizing antibodies, this strategy affords the creation of “supersite transplants”, capable of binding (and potentially eliciting) antibodies similar to the template collection of effective antibodies. Here we transplant three supersites of HIV-1 vulnerability, each targeted by effective neutralizing antibodies from multiple donors. To implement our strategy, we chose a single representative antibody against each of the target supersites: antibody 10E8, which recognizes the membrane-proximal external region (MPER) on the HIV-1 gp41 glycoprotein; antibody PG9, which recognizes variable regions one and two (V1V2) on the HIV-1 gp120 glycoprotein; and antibody PGT128 which recognizes a glycopeptide supersite in variable region 3 (glycan V3) on gp120. We used a structural alignment algorithm to identify suitable acceptor proteins, and then designed, expressed, and tested antigenically over 100-supersite transplants in a 96-well microtiter-plate format. The majority of the supersite transplants failed to maintain the antigenic properties of their respective template supersite. However, seven of the glycan V3-supersite transplants exhibited nanomolar affinity to effective neutralizing antibodies from at least three donors and recapitulated the mannose_9_-*N*-linked glycan requirement of the template supersite. The binding of these transplants could be further enhanced by placement into self-assembling nanoparticles. Essential elements of the glycan V3 supersite, embodied by as few as 3 *N*-linked glycans and ∼25 Env residues, can be segregated into acceptor scaffolds away from the immune-evading capabilities of the rest of HIV-1 Env, thereby providing a means to focus the immune response on the scaffolded supersite.

## Introduction

Antibody isolation and immunogen design are critical components in the current global effort to design an effective vaccine against the human immunodeficiency virus type 1 (HIV-1) (reviewed in [1], [2]). The HIV-1 viral spike is metastable and protected from antibody recognition by multiple overlapping mechanisms of immune evasion [3]–[8]. A plethora of antibodies are elicited against the viral spike or its component subunits, gp120 and gp41, early in natural infection or after vaccination, but virtually none are capable of broad and potent neutralization. Nevertheless, after 2–3 years, the human immune system does generate – in ∼20% of HIV-1-infected individuals – effective broadly neutralizing HIV-1 antibodies [9], [10], with about half of chronically infected donors showing antibodies of neutralization breadth [11]–[16]. Substantial effort has been mounted to isolate such antibodies (to understand their immune mechanisms of effective HIV-1 recognition) and to elicit similar antibodies (to protect prophylactically against HIV-1 infection). The difficulty is that, in sera, broadly neutralizing antibodies exist among a much larger number of Env-reactive antibodies with poor or no neutralizing capacity, and immunization with Env variants has not succeeded in eliciting broadly protective responses. There is thus a need for probes to bind specifically to desired effective antibodies and for immunogens to elicit them.

**Figure 1:**
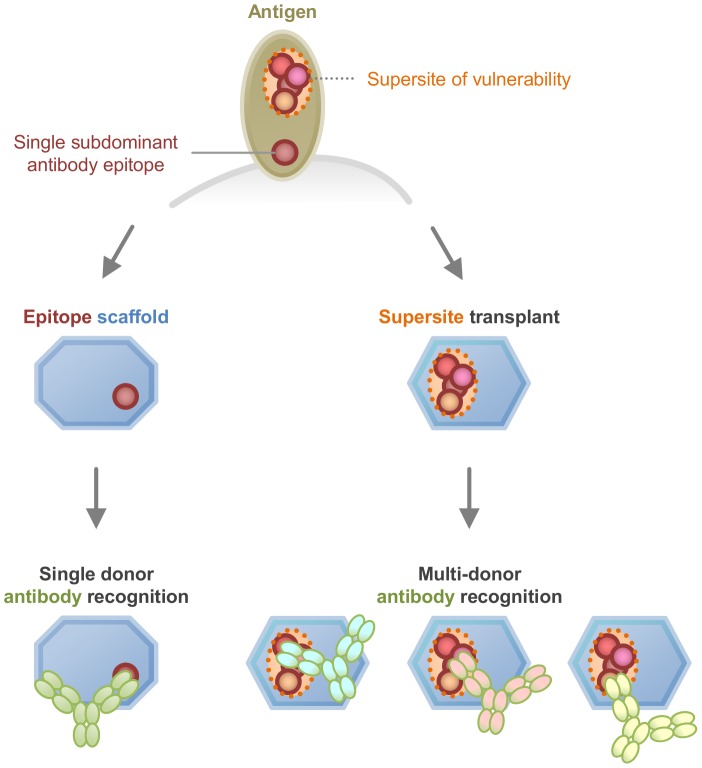
Supersite transplants. Select locations of an antigen may comprise supersites of vulnerability, each of which is comprised of the overlapping epitopes of effective neutralizing antibodies that arise commonly in multiple donors as a result of the immune response to natural infection. One such site, surrounded by a dotted outline with epitopes shown as colored circles, is depicted at top. Supersite transplantation involves the placement of the supersite into a protein scaffold capable of preserving recognition to broadly neutralizing antibodies, elicited in multiple donors (right). By contrast, epitope scaffolds involve the transplantation of an epitope targetted by a single rare or subdominant antibody (left).

HIV-1 Env contains many immunodominant epitopes recognized by non-neutralizing antibodies, which greatly outnumber the desired effectively neutralizing antibodies. One strategy to circumvent this problem involves the development of antibody-epitope scaffolds [17]–[23], in which the residues recognized by a template antibody are separated from the rest of Env through transplantation to a heterologous protein scaffold, while retaining high affinity to the template antibody (**Fig. 1** **, left**). However, because the immune system generates a highly diverse immune response in each individual, an epitope scaffold capable of only binding to a very small proportion of neutralizing antibodies may be too specific to identify antibodies in diverse sera or to elicit an effective immune response. Rather, a focus on antigenic mimics of supersites of Env vulnerability, sites on the virus recognized by multiple highly effective neutralizing antibodies from multiple infected donors (reviewed in [24]), might recapitulate the ability of the template supersite to be recognized by antibodies from diverse sera and to elicit similar effective antibodies upon inoculation (**Fig. 1** **, right**).

Here we present a supersite-transplantation strategy that (1) used a structural alignment algorithm to identify acceptor scaffolds, (2) employed a 96-well expression platform to facilitate experimental screening of transplants, and (3) validated transplantation with broadly neutralizing antibodies from multiple donors. We screened three HIV-1 supersites of vulnerability to neutralizing antibody. With each of the three sites, we and others had previously determined at least one broadly neutralizing antibody bound to the target supersites. Our results demonstrate that a scaffolding-transplantation algorithm, which allows for a limited degree of structural variation in epitope matching, can identify proteins with suitable backbone geometry to accommodate a site of interest, and that a combined computational-experimental strategy offers an efficient method for the design and screening of probes and immunogen candidates. Furthermore, we provide insight into the glycan V3 supersite [25], perhaps the region of HIV-1 Env most commonly recognized by broadly neutralizing antibodies elicited in the first 2–3 years of infection. Notably, as little as 25 HIV-1 Env amino acids – when properly scaffolded – are able to recapitulate much of the antigenicity of this important Env region of HIV-1 vulnerability to neutralizing antibody.

## Results

### Supersite transplantation

The essence of supersite transplantation or scaffolding strategies lies first in the assumption that there exist a sufficient number of proteins in the structure database suitable for presenting the epitope or Env site of interest, and second in the assumption that the antigenic properties of a target supersite – which may encompass a relatively large portion of Env – can be recapitulated in a structurally constrained subportion of the template site. As shown in recent studies [17]–[23], generally only a few scaffolded epitopes have the desired antigenic profile, likely due to the small number of scaffolds obtained or to expression problems with epitope scaffolds caused by the grafting of a foreign epitope.

We used TM-align [26], a structural alignment algorithm, to search for suitable scaffolds in a culled database of protein structures (**Fig. S1**). In contrast with other widely used methods [27], [28], TM-align uses a scoring scheme that emphasizes global similarity in structure over local deviations as is typically employed when using root-mean-square deviation (RMSD). Since our ultimate goal was to graft the transplanted region onto a selected scaffold, we used a steric clash filter with van der Waals and DFIRE statistical potentials [29] to ensure that selected scaffolds could accommodate the transplanted region after grafting.

TM-align-identified scaffolds were evaluated for their fitness to accept the HIV-1 transplant based on RMSD, clash score, and a solvent accessibility ratio of greater than 40 after transplantation (see Methods). The next sections provide details on transplantation of three supersites of HIV-1 vulnerability: the membrane-proximal external region (MPER) on HIV-1 gp41; variable regions 1 and 2 (V1V2) on the HIV-1 gp120 envelope glycoprotein, and the conserved glycan base of variable region 3 (glycan V3) also on gp120.

### MPER-supersite transplants

**Figure 2:**
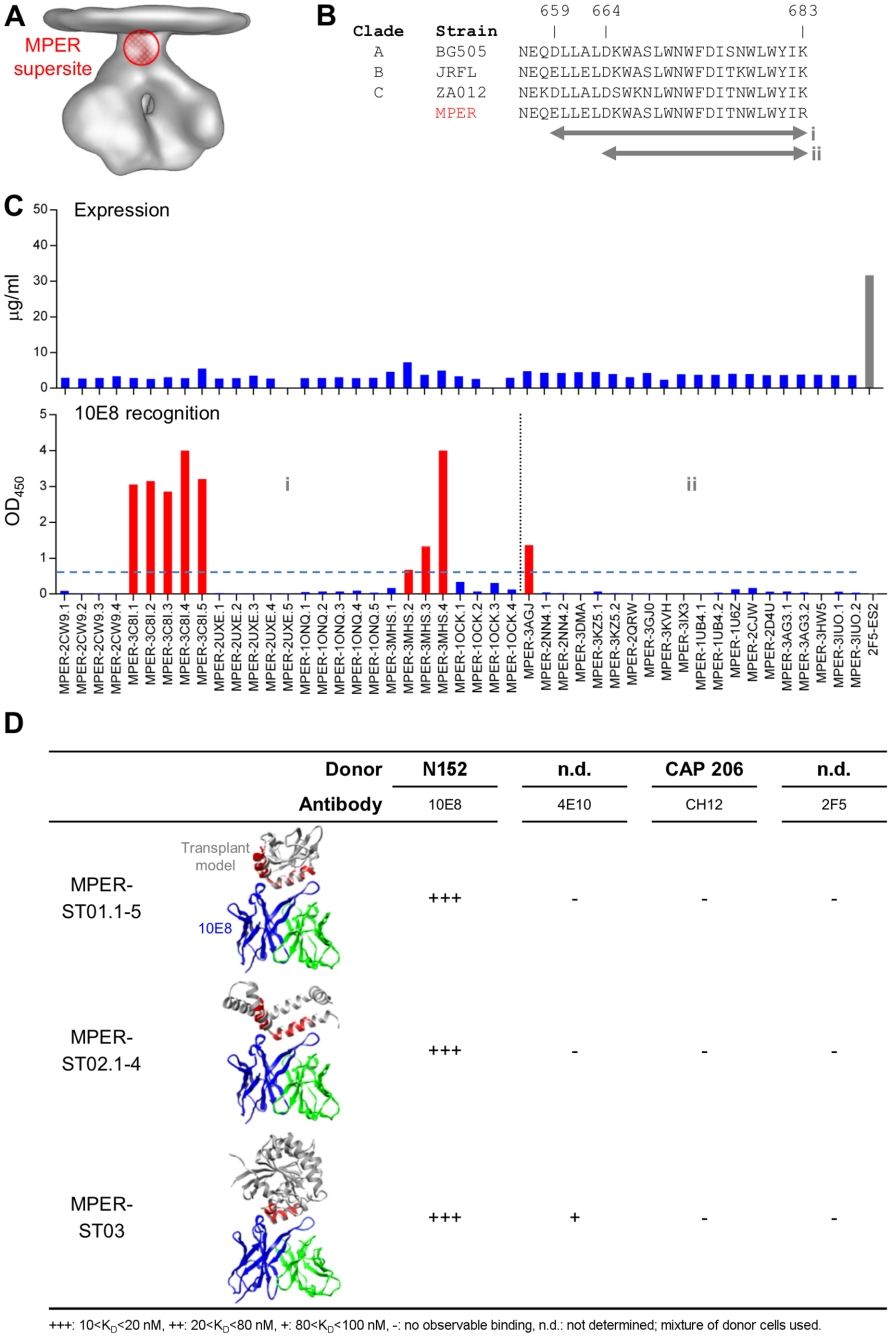
MPER-supersite transplants. Supersite transplants were designed to maintain the 10E8 epitope in its antibody bound conformation. (**A**) Location of MPER supersite on HIV-1 viral spike. (**B**) Sequence alignment of MPER sequences from HIV-1 clades A, B, and C, covering residues 656–683. Boxes shown represent subregions of the MPER supersite that were transplanted. (**C**) 96-well expression and 10E8 antibody binding of 42 site transplants. 2F5-ES2 was used as expression control. (**D**) Superposition of 3 best site transplants (grey) with the 10E8 MPER epitope (red), and the variable domains of 10E8 (heavy chain: blue; light chain: green). The full epitope (residues 659–683) is shown for MPER-ST02 and MPER-ST05, whereas predominantly the turn and C-terminal helix (residues 664–683) for MPER-ST07, for which the full N-terminal helix is not matched. Reactivity of the site transplants with antibodies from different donors is shown.

Antibodies recognizing the membrane-proximal external region (MPER) on gp41 (**Fig. 2a**), such as 2F5 [30] and 4E10 [31], [32] were among the earliest broadly neutralizing HIV-1 antibodies isolated. The more recent isolation of the MPER-specific antibody 10E8 [33] – which neutralizes >98% of tested viruses, shows no signs of autoreactivity, and binds to an epitope that overlaps that of 4E10 – confirms the MPER as an HIV-1 supersite of vaccine interest. The structure of 10E8 in complex with a peptide encompassing the entire 28-residue gp41 MPER (residues 656-683) was previously determined at a resolution of 2.1 Å [33]. The structure of 10E8-bound MPER was found to be comprised of two helices, with the C-terminal helix forming the primary contacts with 10E8 and the hinge region between the two helices forming additional contacts with the second complementarity-determining region of the 10E8 heavy chain (CDR H2). Structural analysis and binding experiments revealed that the minimal 10E8 epitope is likely located within residues 668–683 of the MPER peptide [33], which overlaps considerably with the 4E10, Z13, and CH12 epitopes [34]–[36].

Through use of the structural alignment algorithm, two definitions of the 10E8-defined target site were explored to search for suitable acceptor scaffolds: one comprising gp41 residues 659–684 as in chain F in the crystal structure (PDB ID 4G6F) [33], and the other comprising gp41 residues 664–684, composed predominantly of the C-terminal helix and hinge regions as in chain P (**Fig. 2b**). The C_α_ RMSD cutoff used in epitope matching was set to 3 Å in the first scaffolding search and to 2 Å in the second search. Six scaffolds were identified with backbone geometry resembling the full 10E8 epitope (**Table 1**), and for each of these, 4–5 variants were designed to graft the minimal epitope, the minimal epitope plus hinge, and the full epitope (**Table S1 in File S1**). In total, 27 MPER-supersite transplants were obtained from the search using the full epitope. For the search using only the C-terminal helix and hinge region of the epitope, 15 parent scaffolds, or 20 including variants thereof, were obtained (**Table 1**). The 47 transplants from both searches were expressed in HEK293T cells in a 96-well microplate format, with a 2F5 scaffold (2F5-ES2) [17] used as control. Expression levels were quantitated by bio-layer interferometry using anti-penta-his biosensors. Overall the MPER-supersite transplants showed low expression, most likely attributed to the exposed hydrophobic surface (**Fig. 2c** **, top panel**). When tested for 10E8 binding, three parent supersite transplants derived from PDB IDs 3C8I, 3MHS [37] and 3AGJ [38], heretofore referred to as MPER-ST01, MPER-ST02, and MPER-ST03, respectively, showed high reactivity in ELISA assays (**Fig. 2c** **, bottom panel**), suggesting that the 10E8 epitope was indeed present in these transplants. Two of the transplants, MPER-ST01 and MPER-ST02, were identified in searches with gp41 residues 659–683 and yielded predicted C_α_-RMSDs of 1.34 and 1.25 Å to the gp41 template, respectively (**Table 1**). Similarity of epitope-matching regions was particularly high for the primary 10E8 contact region within the C-terminal helix, yielding C_α_-RMSDs of 1.28 and 0.80 Å (with the full epitope used for superposition), respectively. The third transplant recognized by antibody 10E8, MPER-ST03, was identified in searches with the more limited MPER epitope (gp41 residues 664–683) (**Table 1**).

**Table 1:** Twenty-one acceptor proteins identified for MPER-supersite transplantation by using a TMalign-based search method.^a^

Acceptor (PDB ID)	MPER-supersite transplant	Chain	SS% (H/S)	N_res_	N_ali_	C_α_-RMSD	TM-score	SA ratio	Clash	N_var_
2CW9	-	A	41.2/32.4	182	28	1.43	0.237	0.630	3.841	4
3C8I	1	A	18.9/37.0	127	26	1.34	0.300	0.657	1.321	5
2UXE	-	A	69.3/0.0	114	26	1.06	0.332	0.550	4.068	5
1ONQ	-	A	24.5/43.1	274	26	1.29	0.161	0.508	2.868	5
3MHS	2	B	82.4/0.0	91	26	1.25	0.380	0.592	0.000	4
1OCK	-	A	38.3/15.0	412	26	1.05	0.115	0.527	6.285	4
3AGJ	3	A	18.8/32.4	432	19	1.40	0.080	0.505	0.000	1
2NN4	-	A	69.0/0.0	62	19	1.73	0.386	0.526	0.000	2
3DMA	-	A	39.4/17.6	340	19	1.40	0.101	0.503	0.000	1
3KZ5	-	A	25.5/38.3	47	19	1.21	0.455	0.663	0.000	2
2QRW	-	A	67.5/0.0	126	19	1.53	0.229	0.529	1.199	1
3GJ0	-	A	29.5/24.6	207	19	1.50	0.152	0.521	1.396	1
3KVH	-	A	31.8/30.2	192	20	1.76	0.168	0.504	2.516	1
3IX3	-	A	47.9/16.6	163	19	1.83	0.178	0.457	2.526	1
1UB4	-	A	25.2/34.0	103	19	1.71	0.253	0.499	0.000	2
1U6Z	-	A	45.2/15.7	498	20	0.89	0.076	0.408	2.441	1
2CJW	-	A	39.3/22.5	178	19	1.64	0.168	0.643	1.120	1
2D4U	-	A	85.0/0.0	167	19	1.32	0.185	0.504	0.000	1
3AG3	-	H	54.0/0.0	79	20	1.31	0.345	0.618	0.000	2
3HW5	-	A	50.8/13.0	177	19	1.37	0.174	0.406	2.312	1
3IUO	-	A	70.9/0.0	110	21	1.83	0.274	0.538	2.398	2

a Listed items include supersite index, PDB identifier, chain name, percent secondary structure composition (helix/sheet), number of residues in the supersite, number of residues aligned to the epitope, C_α_ RMSD of aligned residues, TM-score from TMalign output, solvent accessibility ratio between the epitope-matching region in the transplant context versus the epitope alone, transplant-antibody clash score, and number of variants designed based on each transplant.

MPER-supersite transplants recognized well by antibody 10E8 were next tested for recognition by MPER-specific antibodies from other donors, including antibodies 4E10 and CH12, whose epitopes, like that of 10E8, are located within the C-terminal portion of the MPER [31], [36] (**Fig. 2d**). Three of the MPER-supersite transplants, MPER-ST01-03, showed recognition to both 10E8 and 4E10, but binding to 4E10 was noticeably weaker than to 10E8 and no recognition to CH12 was observed. In contrast, the MPER as a free peptide is known to bind all MPER-directed broadly neutralizing antibodies with high affinity [33], [36], [39].

### V1V2-supersite transplants

**Figure 3:**
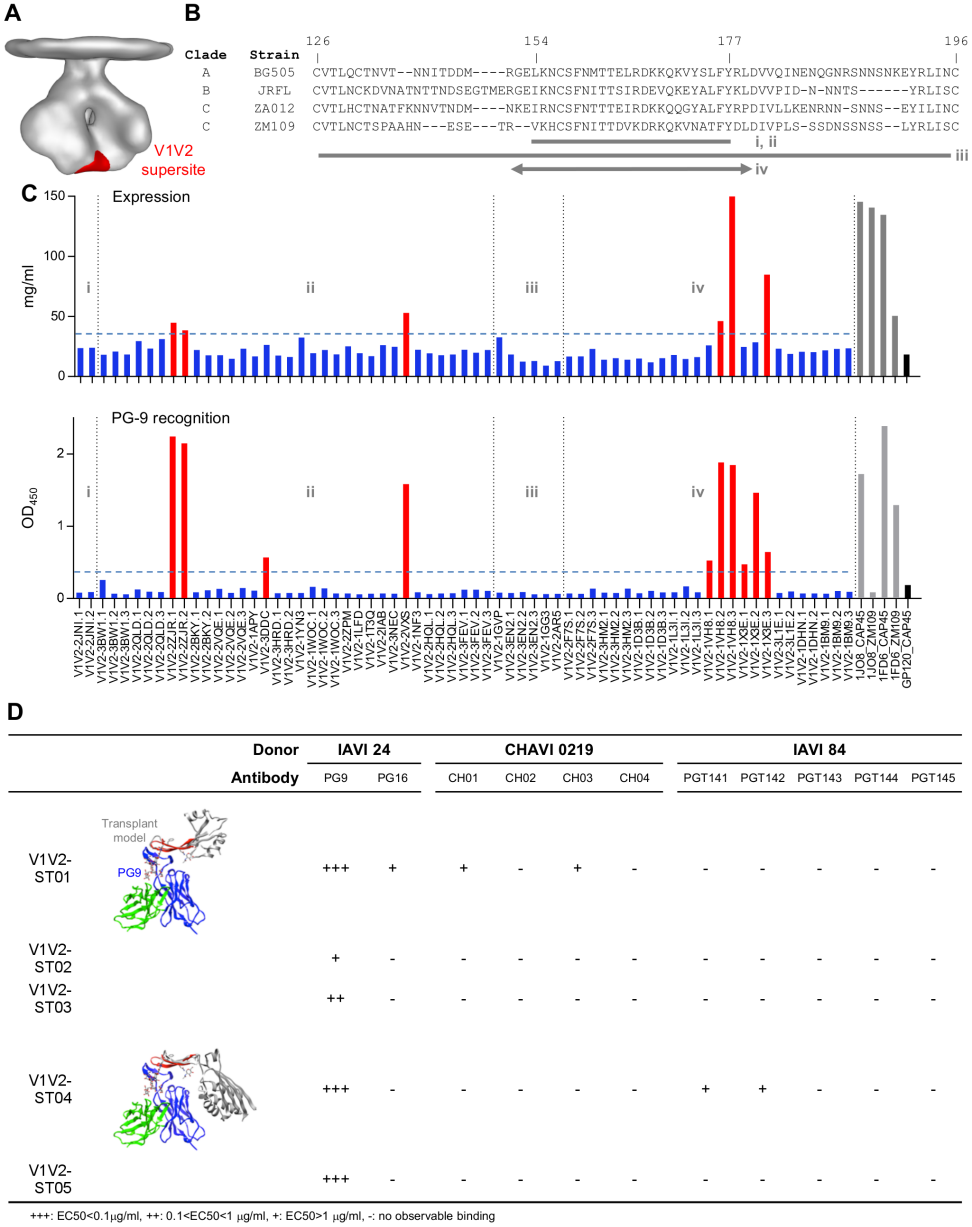
V1V2-supersite transplants. Supersite transplants were designed to maintain the PG9-interacting strands B and C in the antibody bound conformation. Four design ideas were explored for transplanting the B and C strands: matching (i) strands B and C (residues 154–177) to small structurally characterized peptides; (ii) strands B and C to surface-exposed β-hairpin regions; (iii) the entire Greek key motif (residues 126–196) comprising four strands to surface-exposed patches; and (iv) extending the base of the B and C strands (**A**) V1V2-supersite location on viral spike. (**B**) Sequence alignment of the V1V2 domain from three clades and the transplanted strain ZM109. Regions of V1V2 used for transplant design are marked below. (right) The location of the supersite on the viral spike is displayed in green. (**C**) (top) 96-well expression of 67 V1V2 transplants and Ni-Sensor Octet quantitation of expression levels. Four full V1/V2 domain transplants: 1JO8_CAP45, 1JO8_ZM109, 1FD6_CAP45 and 1FD6_ZM109 (grey) and a monomeric gp120 (black) were used as expression controls. (bottom) PG9 ELISA of 67 V1V2 transplants. 10 of the PG9-epitope scaffolds showed substantial interaction with PG9 (red). (**D**) V1V2-supersite transplants which showed significant PG9 binding were tested for binding to additional V1V2-directed antibodies with two displaying weak binding to antibodies from different donors.

Although variable regions 1 and 2 (V1V2) of the HIV-1 gp120 envelope glycoprotein are among the most sequence variable regions of the HIV-1 viral spike, V1V2 can be recognized by a number of glycopeptide-directed broadly neutralizing antibodies such as PG9, PG16, CH01-04 and PGT141–145, from donors IAVI 24, CHAVI 0219 and IAVI 84, respectively [40]–[42] (**Fig. 3a**). One particular *N*-linked glycan at position 160 (HXB2 numbering) defines specificity for all these antibodies, with a second glycan at residue 156 or 173 (depending on isolate) also of importance. The structure of the V1V2 domain in complex with antibody PG9 has been determined for two clade C strains (CAP45 and ZM109) to 2.19 and 1.80 Å, respectively, delineating atomic details of its glyco-peptide epitope [43]. These structures reveal that V1V2 adopts a Greek key motif with strands B and C harboring two PG9-interacting glycans. Similar results were also seen in the structure of V1V2 in complex with PG16, which is clonally related to PG9, although the specific glycan requirements differed [44]. The structure of PG9 bound to a soluble gp140 trimer revealed an asymmetric recognition suggesting that potential quaternary interactions may account for the preference of viral spikes over monomeric gp120s for PG9 [45], and the recent structures of the BG505 SOSIP trimer confirmed atomic-level details for a quaternary V1V2 epitope to be spatially in close proximity to the trimeric axis of the viral spike [7], [8]. Nonetheless, PG9 has sufficient affinity to monomeric full length V1V2 scaffolds to form stable complexes, with the primary interaction mediated through a glycopeptide epitope from strands B and C of a single gp120 protomer [43].

We tested whether the transplantation of less than the full trimeric V1V2 – perhaps as little as monomeric strands B and C – might allow for a site transplant with antigenic properties comparable to the entire V1V2 (**Fig. 3b**). By using a structural alignment-based scaffolding algorithm, four design “ideas” were explored by matching (i) strands B and C (residues 154–177) to small structurally characterized peptides; (ii) strands B and C to surface-exposed β-hairpin regions; (iii) entire Greek key motif (residues 126–196) comprising four strands to surface-exposed patches; and (iv) an extended stem of the B and C strand termini to similar regions in proteins that can be used as a base to graft strands B and C. A search of the protein structure database identified 33 scaffolds as candidates for site transplantation (**Table 2**) and 67 supersite transplants consisting of strands B and C from V1V2 were designed with up to 3 variants per individual scaffold (**Table S2 in File S1**).

**Table 2:** Thirty-three protein scaffolds identified for PG9 epitope transplantation using a TMalign-based search method.^a^

Acceptor (PDB ID)	V1V2-supersite transplant	Chain	SS% (H/S)	N_res_	N_ali_	C_α_-RMSD	TM-score	SA ratio	Clash	N_var_
2JNI	-	A	0.0/76.2	21	21	0.94	0.385	0.881	0	2
3BW1	-	A	6.1/51.2	86	22	1.12	0.34	0.76	0	3
2QLD	-	A	10.5/40.9	171	24	0.93	0.233	0.749	3.169	3
2ZJR	1	Q	20.4/17.2	93	22	1.76	0.298	0.711	1.342	2
2BKY	-	A	31.5/41.6	89	22	1.32	0.326	0.697	0	2
2VQE	-	Q	11.5/51.9	104	24	1.64	0.303	0.694	10.274	3
1APY	-	B	20.6/33.3	141	22	1.61	0.227	0.669	3.946	1
3DDC	2	B	24.1/39.1	133	23	1.77	0.24	0.668	4.539	1
3HRD	-	D	31.9/16.2	160	22	1.94	0.197	0.65	0	2
1YN3	-	A	22.4/42.9	98	23	1.84	0.291	0.642	1.184	1
1WOC	-	A	0.0/68.7	99	22	1.49	0.304	0.635	0	3
2ZPM	-	A	16.3/36.0	86	22	1.3	0.328	0.616	0	1
1LFD	-	A	17.2/33.3	87	24	2.13	0.319	0.693	3.157	1
1T3Q	-	A	31.5/15.4	162	22	2.18	0.196	0.666	1.066	1
2IAB	-	A	20.9/38.6	153	24	2.07	0.218	0.662	3.823	1
3NEC	-	A	25.3/41.4	162	22	2.52	0.175	0.66	0	1
2VXS	3	A	4.7/47.7	86	24	2.44	0.293	0.658	0	1
1NF3	-	C	12.2/38.2	123	22	2.03	0.234	0.646	0	1
2HQL	-	A	10.6/57.7	104	24	2.35	0.269	0.613	52.008	3
3FEV	-	A	0.0/56.9	65	23	2.27	0.332	0.607	6.484	3
1GVP	-	A	0.0/46.0	87	39	2.9	0.412	0.599	0	1
3EN2	-	A	6.6/64.8	91	39	3.29	0.359	0.525	0	3
1GG3	-	A	33.0/17.9	279	38	3	0.186	0.524	2.127	1
2AR5	-	A	35.9/20.5	117	40	2.86	0.36	0.517	9.193	1
2F7S	-	A	34.6/25.7	179	26	1.42	0.223	0.784	2.127	3
3HM2	-	A	31.0/28.1	171	27	1.18	0.249	0.677	4.71	3
1D3B	-	B	0.0/56.8	81	25	1.94	0.347	0.665	0	3
1L3I	-	A	31.7/29.6	186	27	1.53	0.221	0.624	2.127	3
1VH8	4	A	29.4/36.6	153	27	2.49	0.234	0.735	2.127	3
1X3E	5	A	8.2/50.0	110	26	2.35	0.269	0.619	0	3
3L1E	-	A	0.0/50.0	105	27	2.64	0.255	0.616	0	2
1DHN	-	A	32.2/33.1	121	26	2.26	0.272	0.61	2.127	2
1BM9	-	A	54.3/11.7	120	26	2.42	0.279	0.605	27.675	3

a Listed items include scaffold index, PDB identifier, chain name, percent secondary structure composition (helix/sheet), number of residues in the scaffold structure, number of residues aligned to the epitope, C_α_ RMSD of aligned residues, TM-score from TMalign output, solvent accessibility ratio between the epitope-matching region in the scaffold context versus the epitope alone, scaffold-antibody clash score, and number of variants designed based on each scaffold.

All 67 supersite transplants were expressed in HEK293 GnTI^−^ cells in a 96-well microplate and expression levels were quantitated by bio-layer interferometry using anti-penta-his biosensors. Four scaffolded V1V2 proteins with high expression level, 1JO8_Cap45, 1JO8_ZM109, 1FD6_Cap45 and 1FD6_ZM109 [43], were used as controls in the expression assays with a full length gp120 lacking a his-tag as a negative control. Overall, the expression level of the 67 supersite transplants was lower than the four scaffolded V1V2 proteins by several-fold; however, several site transplants showed comparable expression levels (**Fig. 3c** **, upper panel**). The transplants were further examined for PG9 binding via ELISA with Ni^2+^-coated plates. Although most of the proteins showed negligible binding to PG9, epitope-scaffolds derived from 2ZJR [46], 2VXS [47], 1VH8 [48], 3DDC [49] and 1X3E [50] exhibited similar PG9 binding reactivity (by OD_450_) with respect to the four scaffolded full length V1V2 domains (**Fig. 3c** **, lower panel**), suggesting that the PG9-bound epitope conformation was properly presented in these scaffolds while employing only strands B and C from the V1V2 domain.

In addition to PG9 binding, all supersite transplants were tested for binding to broadly neutralizing V1V2-directed antibodies from other donors, such as PGT142 and PGT145 (donor IAVI84) and CH01 and CH04 (donor CHAVI 0219) to validate appropriate site transplantation (**Fig. S2**). ELISA results reveal that (1) whereas the scaffolded V1V2 proteins used in the crystallization of PG9 [43] only bound to PG9, two supersite transplants (V1V2-ST01 and V1V2-ST04) that bound to PG9 were also reactive to other V1V2-directed neutralizing antibodies (**Fig. 3d**); and (2) although the same minimal epitope was presented by each scaffold, specific V1V2-directed antibodies bound differently depending upon the scaffolding context, with PGT142 showing the broadest recognition. Despite ELISA-positive binding, none of these transplants retained the nanomolar affinity of the trimeric V1V2 “cap” to broadly neutralizing V1V2-directed antibodies, indicating a failure of these transplants to recapitulate the antigenicity of the target supersite. Likely this failure relates to the quaternary requirements of most V1V2-directed antibodies, and the fact that only a monomeric V1V2 was transplanted in the supersite transplants described here.

### Glycan V3-supersite transplants

**Figure 4:**
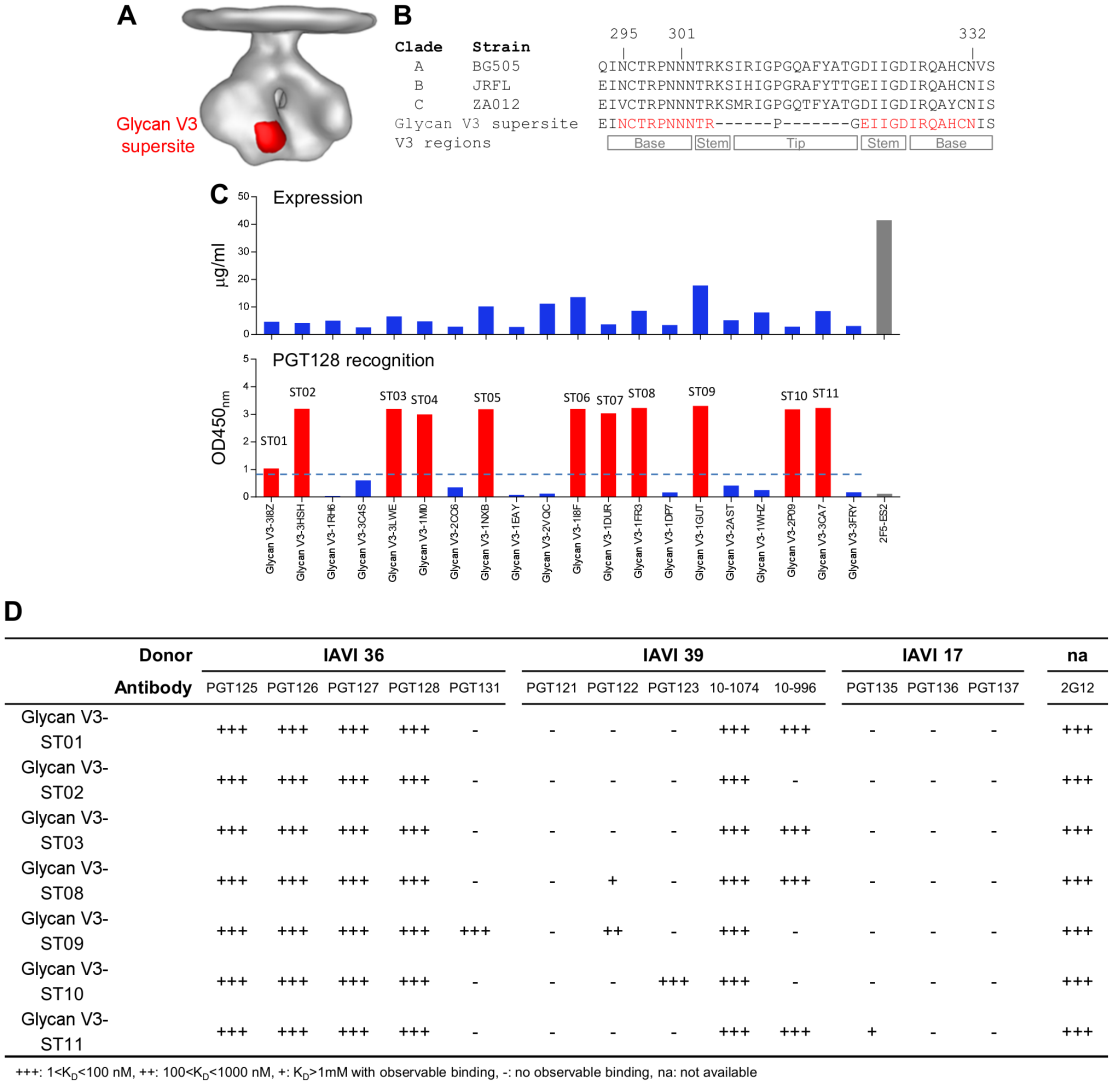
Glycan V3-supersite transplants. Supersite transplants were designed to maintain the mini-V3 in its PG128-bound conformation. (**A**) Location of glycan V3 supersite on HIV-1 viral spike. (**B**) Sequence alignment of the V3 loop from clades A, B and C with potential glycosylations positions marked above and V3 region-structural elements marked below. The transplanted glycan V3-supersite sequence was shown with the mini-V3 highlighted in red. (**C**) (top) 96-well expression of 20 glycan V3-supersite transplants and Ni-Sensor Octet quantitation of expression levels. 2F5-ES2 was used as expression control. (bottom) ELISA of PGT128 antibody binding of the expressed glycan V3 transplants. 11 of the 20 glycan V3 transplants showed substantial interaction with PGT128 (red). (**D**) Glycan V3-supersite transplants which showed significant PGT128 binding were tested for binding to additional glycan V3-directed antibodies from different donors.

A glycosylated conserved portion of the variable region 3, termed “glycan V3” on HIV-1 gp120, is another vaccine target of importance (reviewed in [24]) (**Fig. 4a**); notably, the glycan V3 supersite is recognized by the most common broadly neutralizing antibodies elicited during the first two to three years of infection. A series of human antibodies, represented by 2G12, PGT121–123, 10–1074, PGT125–131, PGT135–137, and VRC24 have been isolated with glycan V3-directed specificity [42], [51]–[53]. Previously, a truncated V3 region comprising a “mini-V3” [54] of 3 *N*-linked glycan and ∼25 amino acids was developed by Stanfield, Wilson and colleagues in the context of an engineered gp120 outer domain (eODmV3) that bound the PGT128 antibody, and the structure of the complex was reported at a resolution of 3.25 Å (**Fig. 4b**). The crystal structure revealed that PGT128 interacts with two conserved glycans at positions 332 and 301 and with the C-terminal region of the V3 loop, and that a mannose_9_-*N*-linked glycan was critical at position 332 [54]. Based on the structure, we hypothesized that a protein with a surface-exposed β-hairpin that was structurally similar to the mini-V3 would be a suitable acceptor scaffold for the entire glycan V3 supersite of vulnerability. With the structural alignment-based algorithm, two stem regions of the V3 loop were used as templates to search for scaffolds with: (1) number of residues in the range of 40–80, (2) C_α_ RMSD lower than 1.5 Å, and (3) a clash score near zero. Twenty non-homologous proteins were selected (**Table 3**), and the mini-V3 was grafted onto each scaffold (**Table S3 in File S1**).

**Table 3:** Twenty protein scaffolds identified for glycan V3-supersite transplantation by using a TMalign-based search method.^a^

Acceptor (PDB ID)	Gycan V3-supersite transplant	Chain	SS% (H/S)	N_res_	N_ali_	C_α_-RMSD	TM-score	SA ratio	Clash	N_var_
3I8Z	1	A	8.0/44.0	50	11	1.16	0.237	0.567	0.000	1
3HSH	2	A	12.5/32.1	56	12	1.48	0.233	0.492	0.000	1
1RH6	-	A	27.8/25.9	55	12	1.26	0.252	0.544	0.000	1
3C4S	-	A	0.0/56.1	57	12	1.38	0.239	0.431	0.000	1
3LWE	3	A	31.1/36.1	61	12	1.38	0.237	0.565	0.000	1
1MI0	4	A	21.3/42.6	61	12	0.91	0.271	0.554	0.000	1
2CC6	-	A	23.4/51.6	64	12	1.42	0.215	0.434	0.000	1
1NXB	5	A	0.0/41.9	62	11	1.33	0.220	0.495	0.000	1
1EAY	-	C	29.9/25.4	67	12	1.21	0.221	0.457	0.000	1
2VQC	-	A	57.1/17.1	70	12	1.38	0.222	0.480	0.000	1
1I8F	6	A	8.5/59.2	71	12	1.46	0.224	0.546	0.000	1
1DUR	7	A	9.1/18.2	55	11	0.99	0.244	0.416	0.000	1
1FR3	8	A	10.4/50.7	67	10	0.52	0.238	0.522	0.000	1
1DP7	-	P	42.1/31.6	76	10	1.35	0.175	0.611	0.000	1
1GUT	9	A	10.4/46.3	67	11	0.85	0.239	0.476	0.000	1
2AST	-	C	15.9/33.3	69	12	0.57	0.275	0.576	0.000	1
1WHZ	-	A	37.1/22.9	70	12	1.30	0.225	0.511	0.000	1
2P09	10	A	36.2/20.3	69	12	1.25	0.226	0.436	0.000	1
3CA7	11	A	12.0/36.0	50	11	1.04	0.250	0.511	0.000	1
3FRY	-	A	27.4/12.3	73	12	1.43	0.208	0.549	0.000	1

a Listed items include scaffold index, PDB identifier, chain name, percent secondary structure composition (helix/sheet), number of residues in the scaffold structure, number of residues aligned to the epitope, C_α_ RMSD of aligned residues, TM-score from TMalign output, solvent accessibility ratio between the epitope-matching region in the scaffold context versus the epitope alone, scaffold-antibody clash score, and number of variants designed based on each scaffold.

Similar to the screening of V1V2-supersite transplants, all 20 glycan V3-supersite transplants were expressed in HEK293T cells in the 96-well microplate and in the presence of 10 ug/ml kifunensine to ensure uniform mannose_9_-*N*-linked glycosylation. A 2F5 scaffold, 2F5-ES2 [17], with high expression level was used as a control in expression assays (**Fig. 4c** **, top panel**). The binding of the glycan V3-supersite transplants to PGT128 was assessed by ELISA. 11 of these 20 transplants bound to PGT128 with OD_450_ ranging from 1.0 to 3.5 (**Fig. 4c** **, bottom panel, Fig. S3**) (glycan V3-ST01-ST11). We also tested binding of these 11 transplants to glycan V3-directed antibodies from other donors. Remarkably, seven of the glycan V3-site transplants showed tight binding to glycan V3-directed broadly neutralizing antibodies from at least three other donors (**Fig. 4d**).

### Structural and glycan specificity of glycan V3-supersite transplants

Because the seven glycan V3-supersite transplants that we identified through our combined computational-experimental strategy differed so substantially in their antigenic behavior from the more than 100 other transplants in their ability to bind broadly neutralizing antibodies from multiple donors, we sought to further characterize the structural and antigenic integrity of these supersite transplants and their recognizing antibodies.

We first explored whether the recognizing antibodies could bind to mini-V3 in a flexible, non-scaffolded context. We linked mini-V3 comprising the 2 sites of *N*-linked glycosylation and ∼25 amino acids with no scaffold, but only a flexible linker, to the constant region (Fc) of an immunoglobulin to create “mini-V3-Fc”. We test the recognition of mini-V3-Fc by a panel consisting of 15 broadly neutralizing antibodies from 5 donors (**Table 4**). None of the broadly neutralizing antibodies directed with glycan V3-specificity bound to the mini-V3-Fc construct (**Table 4**). A substantial proportion of the broadly neutralizing antibodies did, however, show nM affinity to multiple glycan V3-supersite transplants, though not to other regions of Env (**Table 4**). These results indicate that recognition of the transplanted mini-V3 was dependent on the heterologous protein scaffold providing an appropriate structural context.

**Table 4:** Binding affinity of glycan V3-supersite transplants to antibodies isolated from diverse donors.

Epitope		Glycan V3														V3	MPER	V1V2	CD4BS
Donor		IAVI 36					IAVI 39					IAVI 17			NA	NA	N152	IAVI 24	NIAID45
Antibody		PGT125	PGT126	PGT127	PGT128	PGT131	PGT121	PGT122	PGT123	10–1074	10–996	PGT135	PGT136	PGT137	2G12	447–52D	10E8	PG9	VRC01
	Mini-V3-Fc	NB*	NB	NB	NB	NB	NB	NB	NB	NB	NB	NB	NB	NB	NB	NB	NB	NB	NB
	YU2 gp120	5#	11	35	11	NB	21	NB	NB	39	10	18	48	82	15	5	NB	NB	87
Glycan V3-supersite transplant	ST01	4	5	7	5	NB	NB	NB	NB	19	18	NB	NB	NB	18	NB	NB	NB	NB
	ST02	4	7	9	6	NB	NB	NB	NB	46	NB	NB	NB	NB	32	NB	NB	NB	NB
	ST03	6	8	12	7	NB	NB	NB	NB	27	43	NB	NB	NB	21	NB	NB	NB	NB
	ST08	17	13	18	15	NB	NB	>1000	NB	5	3	NB	NB	NB	20	NB	NB	NB	NB
	ST09	11	6	12	4	11	NB	187	NB	2	NB	NB	NB	NB	15	NB	NB	NB	NB
	ST10	13	16	19	5	NB	NB	NB	NB	26	NB	NB	NB	NB	10	NB	NB	NB	NB
	ST11	14	20	19	6	NB	NB	NB	NB	15	17	>1000	NB	NB	11	NB	NB	NB	NB

*: NB: no binding detected, NA: not available.

# : K_D_ (nM).

**Figure 5:**
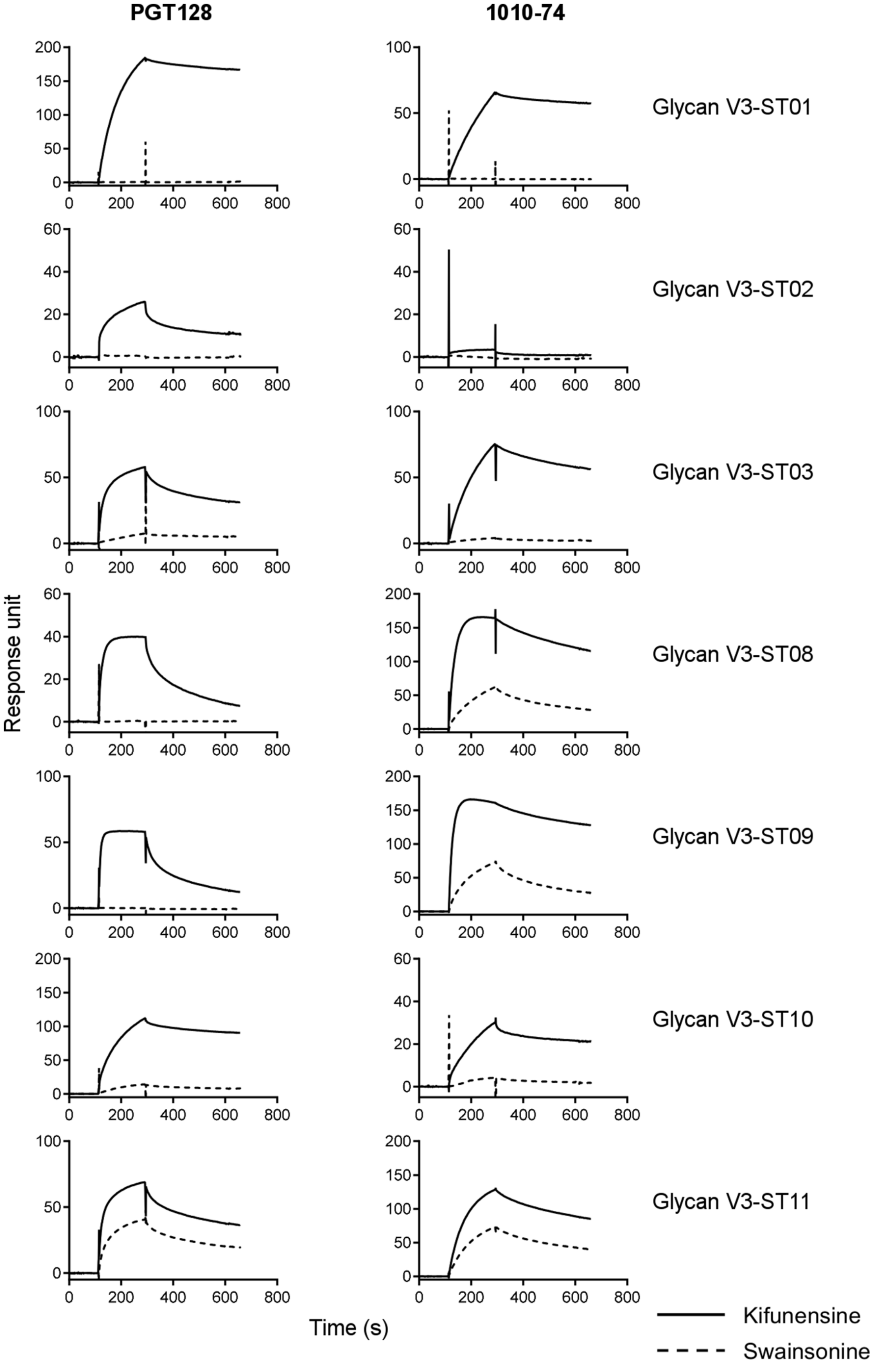
Glycosylation of glycan V3-supersite transplants and antibody reactivity. SPR sensorgrams showed binding of PGT128 (donor 39) and 10–1074 (donor 39) to the 7 glycan V3 transplants expressed in 293F cells in the presence of glycosylation inhibitors kifunensine (solid line) or swainsonine (dashed line).

Next we analyzed the glycan requirements of recognizing antibodies for the glycan V3-supersite transplants. A mannose_9_-*N*-linked glycan is known to be required at residue N332 of HIV-1 for recognition by broadly neutralizing antibodies like PGT122, PGT128, PGT135 and 2G12 [42], [55], [56]. Only when the glycan V3-site transplants were expressed in the presence of kifunensine (which ensures mannose_9_-*N*-linked glycosylation) but not in the presence of swansonine (which allows glycan processing to produce mannose_5_-*N*-linked glycans and hybrid biantennary sugars) did the broadly neutralizing antibodies PGT128, 10–1074 and 2G12 recognize the seven glycan V3-supersite transplants (**Fig. 5**). These results indicate that recognition by the broadly neutralizing antibodies for the glycan V3-supersite transplants retained the same dependence for a particular type of *N*-linked glycosylation as with recognition of native Env.

### Mini-V3 comprises essential components of glycan V3 supersite

**Figure 6:**
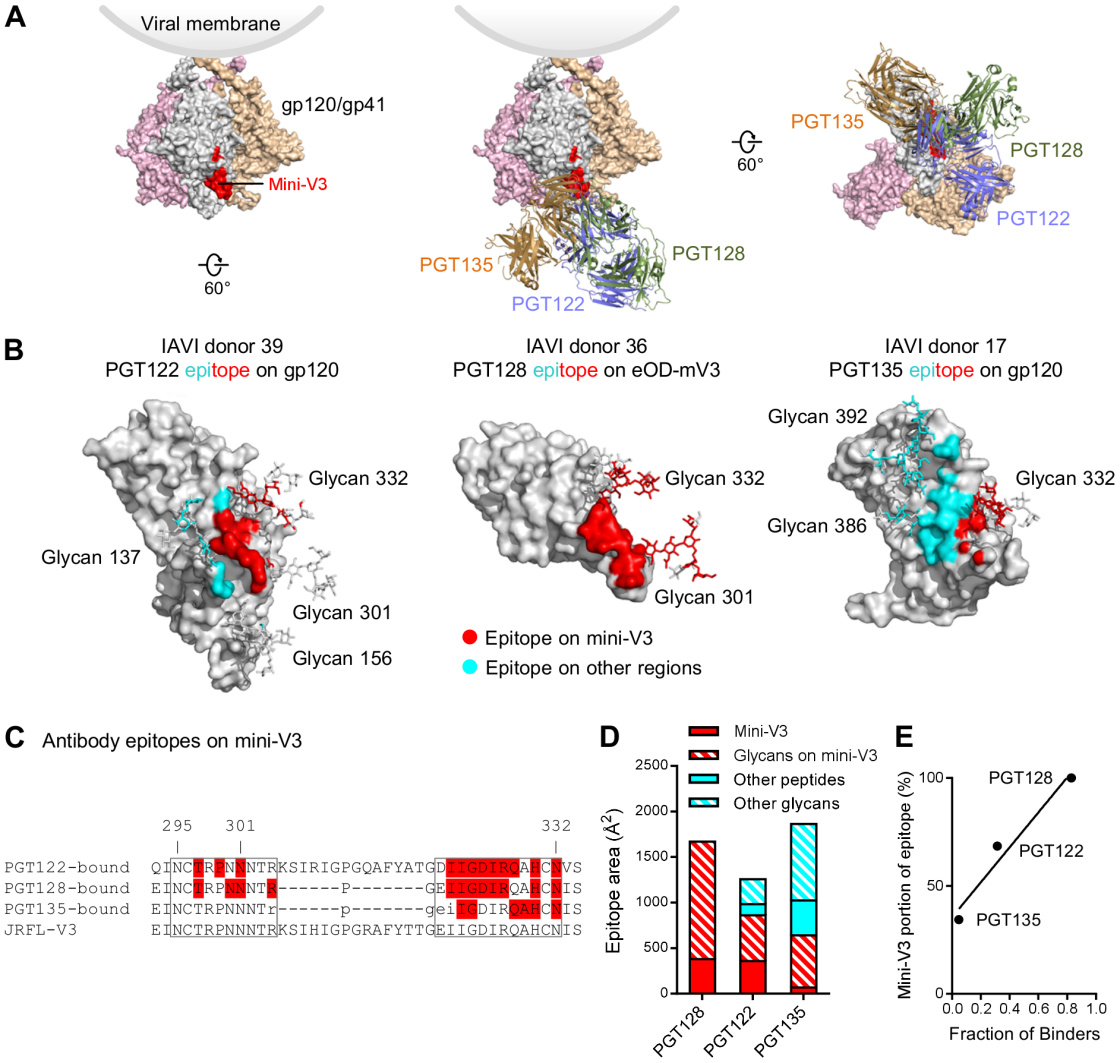
Contribution of the mini-V3 region to the epitopes of antibodies targeting the glycan V3 supersite. (**A**) (left) HIV-1 viral spike with mini-V3 region shown in red. (middle and right) Different views of binding modes for glycan V3-targeting antibodies PGT122, PGT128 and PGT135. (**B**) Surface representation of HIV-1 gp120 with glycans involved in antibody binding shown in sticks. The antibody epitope from the mini-V3 region were colored red and epitope from non-glycan V3 regions were colored cyan. (**C**) Sequence alignment of the glycan V3 region with antibody interacting residues highlighted in red. The mini-V3 was boxed and the residue numbers above marked the potential glycosylation sites. (**D**) Comparison of epitope surface area contributed by the glycan V3 region and other regions for antibodies PGT122, PGT128 and PGT135. (E) Percentage of epitope contribution by transplanted glycan V3 site correlated with the reactivity of the glycan V3-supersite transplants to somatic variants of template antibodies.

To gain insight into the ability of the transplanted mini-V3 to recapitulate both the glycan-requirements and the antigenic recognition of the glycan V3 supersite, we analyzed the structural composition of epitopes recognized by glycan V3-targeting broadly neutralizing antibodies (**Fig. 6a,b**). Crystal structures of Env complexes have been determined with glycan V3-directed broadly neutralizing antibodies from three donors: antibody PGT122 from IAVI donor 39 in the context of the BG505 SOSIP.6R.664 Env trimer [7]; antibody PGT128 from donor IAVI 36 in the context of the gp120 outer domain [54]; and antibody PGT135 from donor IAVI 17 in the context of gp120 core [25]. We analyzed the PGT122, PGT128 and PGT135 recognized epitopes for contribution from the 25 residue mini-V3 (**Fig. 6b–d**). The recognized Env surface of PGT122 comprised about two thirds *N*-linked glycan and one third protein; of these, about 60% of the PGT122 epitope was contained in the mini-V3. For PGT128, the entire recognized Env surface was contained in mini-V3, of which protein surface from about 12 mini-V3 residues contributed 30% of the interactive surface and glycans 301 and 332 contributing the remaining 70% of the interactive surface. For PGT135, less than half the recognized Env surface was contained in mini-V3.

We quantified the relative ability of somatic variants from donors from which multiple broadly neutralizing antibodies have been isolated to recognize the glycan V3-supersite transplants. Antibodies from donor IAVI 36 (PGT125-131) showed the most comprehensive recognition: for the best 7 transplants, 29 tested combinations showed nM affinity out of a possible 35. For donor IAVI 39 (PGT121-123, 10-1074 and 10–996) and with the best 7 transplants, 14 tested combinations showed nM affinity out of a possible 35. For donor IAVI 17 (PGT135–137), for the best 7 transplants, only 1 tested combination showed µM affinity out of a possible 21. Similar ratios of recognition were observed for all 11 transplants with positive ELISA recognition of PGT128. Notably, these ratios correlated with the percentage of the epitope contributed by the mini-V3 (**Fig. 6E**). Altogether these results indicate that the ability of the glycan V3-site transplants to recapitulate recognition by template broadly neutralizing antibodies correlates with the transplanted portion of the epitope. Despite retaining less than 50% of the supersite, glycan V3 transplants may still have nM affinity to the target antibodies.

### Glycan V3-supersite transplants in nanoparticle contexts

**Figure 7:**
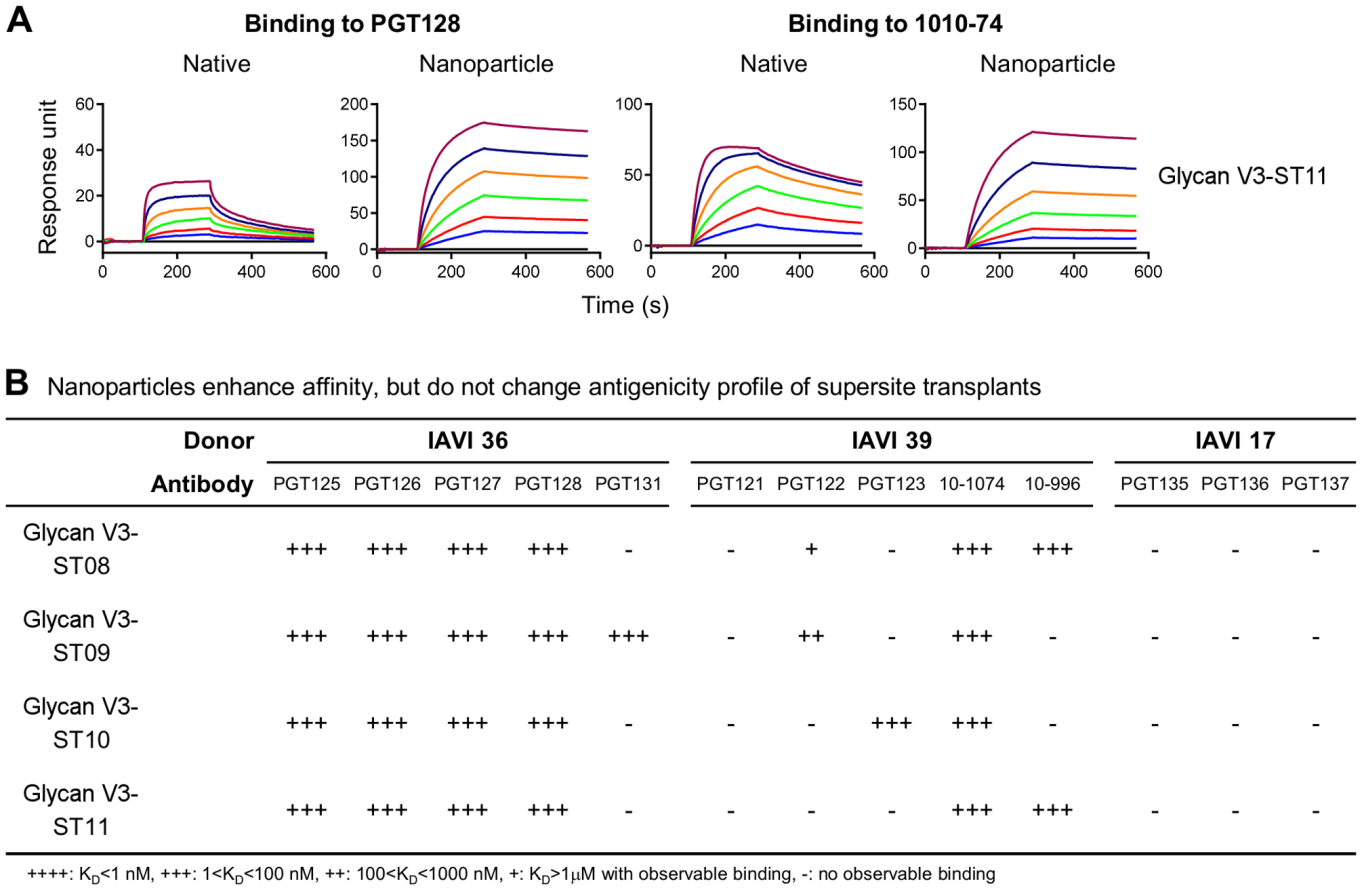
Nanoparticle presentation enhances affinity but not diversity of antigenic recognition of glycan V3-supersite transplants. (**A**) SPR sensorgrams showing the binding to template antibodies PGT128 and 10–1074 for glycan V3 ST11 in its native and ferritin nanoparticle forms. (**B**) Antigenicity profile of glycan V3 ST08-11 in their ferritin nanoparticle forms.

Finally we asked how the placements of the transplants into nanoparticle contexts might alter the recognition by the target antibodies. Because nanoparticles have shown utility in enhancing both antigenicity and immunogenicity [57] and because the small, rigid proteins identified as suitable acceptor scaffolds for the glycan V3-supersite of vulnerability might be also suitable for presentation on a virus-like particle (VLP) or other multivalent platforms [58], we transplanted the glycan V3-supersites into the ferritin nanoparticle context (**Table S4 in File S1**). The ferritin from H. pylori has octahedral symmetry and assembles into a nanoparticle of 24 subunits, arrayed as 8 trimers, and placement of influenza hemagglutinin in this context was shown to enhance immunogenicity by over 10-fold [57]. When tested by surface plasmon resonance, the glycan V3-supersite transplants in the ferritin nanoparticle context retained their ability to bind to broadly neutralizing antibodies. Notably, the off-rate of the interaction with antibodies was reduced in the ferritin nanoparticle context relative to the native immunogen (**Fig. 7a** **,** **Table 5**). We also analyzed how the nanoparticle context affected recognition to the multiple somatic variants from IAVI donors 17, 36 and 39. The results showed that the presentation of glycan V3 site in nanoparticle context did not change the overall antigenic profile although it did improved their apparent affinity (**Fig. 7b**). Together, these results indicate that the antigenicity of mini-V3 could be enhanced by structure-based means of optimization, in this case, by nanoparticle oligomerization.

**Table 5:** Presentation of glycan V3-supersite transplants in a nanoparticle context reduces their apparent rate of dissociation.

Glycan V3	Binding to PGT128						
	Native			Ferritin			
	k_on_ (M^−1^S^−1^, x10^4^)	k_off_ (S^−1^, x10^−4^)	KD (nM)	k_on_ (M^−1^S^−1^, x10^4^)	k_off_ (S^−1^, x10^−4^)	KD (nM)	Fold decrease of k_off_
ST08	61.7	49.8	8.1	36.7	4.3	1.2	11.5
ST09	116.0	95.9	8.3	58.2	3.8	0.7	25.2
ST10	27.0	7.5	2.8	27.0	4.3	1.6	1.7
ST11	31.6	20.4	6.5	26.0	4.7	1.8	4.3

Glycan V3	Binding to 10–1074						
	Native			Ferritin				
	k_on_ (M^−1^S^−1^, x10^4^)	k_off_ (S^−1^, x10^−4^)	KD (nM)	k_on_ (M^−1^S^−1^, x10^4^)	k_off_ (S^−1^, x10^−4^)	KD (nM)	Fold decrease of k_off_
ST08	45.0	18.7	4.2	21.9	2.5	1.1	7.5
ST09	37.1	7.8	2.1	12.0	2.4	2.0	3.3
ST10	1.3	13.8	105	2.3	1.1	4.8	12.5
ST11	8.4	13.4	16	3.1	3.2	11	4.2

## Discussion

HIV-1 and other pathogens employ multiple overlapping mechanisms to evade the immune response. One potential strategy to overcome these immune-evading mechanisms is to extract a particular viral epitope recognized by a potent neutralizing antibody and to present it in a scaffolded context that retains the antigenic character of the epitope, but separates it from the immune-evading mechanisms that hinder the elicitation of effective neutralizing antibodies in the native viral context.

There are now multiple examples of epitope-focused vaccine design in both sequence and various structure-based contexts. In 2006, Chakraborty and colleagues showed that transplantation of the V3 region from HIV-1 gp120 into a thioredoxin scaffold could elicit neutralizing antibodies to the MN strain of HIV-1, though not to Tier 2 neutralization-resistance strains like JR-FL or BAL [59], while later studies showed that a constrained V3 peptide was a more optimal immunogen than a linear one [60]. In 2010, Ofek and colleagues showed that scaffolding of the MPER epitope for the 2F5 antibody into various acceptor scaffolds allowed for structure-specific elicitation, with elicited antibodies able to induce the scaffolded β-hairpin in flexible MPER peptides, which otherwise assume helical conformations [17]. In 2010, Totrov and colleagues showed that placement of the V3 region from HIV-1 gp120 into a multivalent cholera toxin scaffold could, after DNA-prime/protein boost, elicit an immune response that potently neutralized multiple strains of HIV-1 [61]. In 2013, McLellan and colleagues showed that stabilization of a neutralization-sensitive site of vulnerability on the RSV fusion glycoprotein afforded the elicitation of high titers of RSV-neutralizing antibodies [62]. And in 2014, Correia and colleagues created epitope scaffolds that placed the helix-loop-helix region of RSV recognized by the motavizumab antibody into a multivalent context, which elicited RSV-neutralizing antibodies in NHP [23]; surprisingly, the elicited antibodies bound primarily to the loop region of the epitope, not the helical regions recognized by the motavizumab antibody, demonstrating the difficulty in replicating the elicitation of a particular template immune response.

To surmount such difficulties, we propose that epitope-focused vaccine design should focus on supersites of viral vulnerability, targeted by highly effective neutralizing antibodies elicited in multiple donors. These supersite transplants should, moreover, replicate the antigenicity of the template supersite. It was not clear when we began this study, if such supersite transplants could be successfully created. For example, with the CD4-binding site on HIV-1, a supersite of vulnerability recognized by antibodies such as b12, HJ16, VRC01 and others, b12-epitope scaffolds or minimal fragments have been created [19], [63], the resultant epitope scaffolds and minimal fragments, however, were highly specific for antibody b12 [19], [63].

Our work with other HIV-1 supersites of vulnerability failed to mimic the broad antigenicity of both the MPER and V1V2 regions. The MPER assumes different conformations when recognized by MPER-directed broadly neutralization antibodies, and this conformational diversity likely provides an explanation for the lack of broad antigenicity of the MPER-supersite transplants, which were designed to display a particular MPER conformation. We note in this context that the MPER as a free peptide displays broad antigenicity, but the MPER peptide is also recognized by non-neutralizing antibodies, and thus does not have the desired specificity for broadly neutralizing antibodies of an appropriately scaffolded site transplant. Meanwhile the V1V2 site is close to the trimeric axis of the functional viral spike, and all broadly neutralizing antibodies that recognize this site demonstrate more than 100-fold greater affinity for the trimeric Env assembly over monomeric gp120; the quaternary nature of the V1V2 site provides a likely explanation for the failure of monomeric transplants to re-create the broad antigenicity of V1V2.

In contrast, the glycan V3-supersite transplants, which utilize a mini-V3 portion of HIV-1 comprising 3 *N*-linked glycans and ∼25 amino acids, succeeded in replicating the antigenicity of the glycan V3 supersite against a number of broadly neutralizing glycan V3-directed antibodies from diverse donors. Moreover, the mini-V3 in a non-scaffolded context failed to be recognized by broadly neutralizing antibodies, indicating that the structural integrity provided by the scaffold was critical to the glycan V3-supersite transplants.

Only a portion of the glycan V3 supersite was transplanted, and the degree of antigenic mimicry against antibodies from a specific donor correlated with the portion of the transplanted supersite recognized by antibodies from that specific donor (**Fig. 6E**). A design that incorporates the transplantation of a larger portion of the supersite may have greater antigenic breadth. In this regard, it should be noted that the glycan V3-supersite is one of the most promiscuously recognized of the HIV-1 Env supersites of vulnerability. Antibodies that recognize this supersite range from PGT122, which binds a glycopeptide V1-V3 spanning epitope [7], to PGT135, which binds a glycopeptide V3-V4 spanning epitope [25], and to 2G12, which binds a glycan-only epitope [64]. It remains to be seen whether transplants comprising greater portions of the glycan V3 supersite would make more effective probes or immunogens; we note in this context that it remains to be shown whether the glycan V3-supersite transplants described here can elicit effective HIV-1-neutralizing antibodies – either alone, as cocktails, or as components in prime/boost regimens. Nevertheless, the structural and antigenic analyses described here, along with the successful oligomerization of the glycan V3-supersite transplants in the ferritin nanoparticle context, do demonstrate that supersite transplants with antigenic mimicry of the template supersite can be achieved through transplantation of only a portion of the recognized supersite. Overall, the design framework and described glycan V3-supersite transplants provide both conceptual context and initial immunogens for a supersite-focused vaccine effort.

## Materials and Methods

### Protein structure database

A protein structure database was compiled for the scaffolding search. To ensure both coverage and diversity of the obtained scaffolds, the PISCES [65] server was used to generate a representative, non-homologous set of Protein Databank (PDB) entries at the 90% identity level, with a resolution cutoff of 3.0 Å, and an R-factor cutoff of 1.0. As of October 29, 2010, the PISCES server yielded an annotated list of 19,500 peptide chains after screening with sequence similarity and structure quality filters. All protein chains with only C_α_ atoms were marked; duplicate chains were eliminated from each entry in the database compilation; modified amino acids were identified from a look-up table of pre-defined types and renamed accordingly with the modified groups removed; insertions were renumbered so that their residue numbers were in ascending order in the resulting PDB file.

### Scaffolding/transplantation for vaccine design

The scaffolding/transplantation-based vaccine design (**Fig. 1**) consists of identification of supersites of HIV-1 vulnerability to broadly neutralizing antibodies on the pathogen, structural characterization of antibody-epitope interactions, structure-based computational design of supersite transplants, and antigenic and structural analysis of the supersite transplants. The computational scaffolding/transplantation procedure is essential to this vaccine strategy and contains four steps.

In Step 1, an exhaustive search is performed to find scaffold proteins suitable for supersite transplantation. In our study, a scaffolding algorithm based on structural alignment [26] was implemented and tested. The scaffolding search results in a list of PDB entries with key parameters calculated for each entry, including protein size (number of residues), matching criteria (C_α_ RMSD and number of residues matched), template-epitope exposure in the scaffold context, and a simple scaffold-antibody clash score (sum of *r* ^−1^ for all heavy-atom contacts within 1 Å). Desired scaffolds are then selected based on these parameters.

In Step 2, two grafting approaches may be used to produce a supersite transplant model. If all template-antibody epitope residues can find matches in the scaffold protein, ‘side-chain grafting’ is sufficient. However, when the template antibody-epitope cannot be matched to a scaffold region residue by residue, ‘backbone grafting’ has to be utilized to generate the atomic model. In such cases, template antibody-epitope residues – both backbone and side chain atoms – are grafted onto target protein by superposition of selected anchoring points, e.g. N- and C-terminal residues of the epitope loop. The supersite-transplant models may be further relaxed by energy minimization.

In Step 3, supersite transplant models are evaluated using empirical scoring functions and statistical potentials, which have been applied to evaluate homology models and large structure ensemble generated by MD simulation [66]. Briefly, the tabulated soft-core van der Waals (vdW) function can be used to detect steric clashes, while the statistical potential DFIRE [29] is more sensitive to unfavorable side-chain contacts. High scoring residues (e.g. ≥2 DFIRE score) interacting with the grafted supersite may be subjected to visual inspection and further side-chain modeling.

In Step 4, problematic residues are optimized using a side-chain modeling/prediction program that combines a statistical potential (DFIRE), an XYZ side-chain rotamer library [67] and a combinatorial search algorithm named FASTER [68].

### Structural alignment-based scaffolding

We used TM-align [26], a structural alignment algorithm, as core engine for scaffolding search. Compared with other widely used methods [28], TM-align uses a different scoring scheme (termed TM-score [69]) that emphasizes more on global similarity than local deviations, that could affect structure fitting and lead to a large RMSD value. Due to the stochastic nature of the structural alignment algorithm, parameters such as epitope length could have notable effect on the outcome and therefore have been tested in each run to find optimal values. For a multi-segment epitope, different orders of the segments were tried in separate scaffolding runs as the structural alignment algorithm assumes a sequential order of the residues provided in the epitope PDB file. It is also worth noting that each epitope residue is weighted equally in the algorithm and as a result the scaffolds found may be biased towards the larger segment(s) in a multi-segment epitope.

### Construction of plasmids encoding supersite transplants

Sequences for each of the scaffolds from different designs were codon optimized for expression in Homo sapiens, synthesized and cloned into the pJ603 or pVRC8400 mammalian expression vectors (DNA 2.0, CA; Life Technologies, CA). For the 10E8 supersite transplants, Hisx8/Strep or GFP/Hisx8 tags were appended to the C-terminus of the transplants. For the V1V2-supersite transplants, Hisx8/Strep tags were appended to the C-terminus of the transplants. For the glycan V3-supersite transplants, HRV3C cleavage site and Hisx8/Strep tags were appended to the C-terminus of the transplants. To create a flexible, non-scaffolded mini-V3, the sequence EINCTRPNNNTRPGEIIGDIRQAHCNIS was fused to the Fc portion of human IgG1 with a GG linker. A HRV3C cleavage site was also inserted after the GG linker for cleaving the mini-V3 peptide off the Fc carrier when needed. The plasmids were prepared by standard methods (DNA 2.0, CA, or GeneImmune, MD).

### Transient transfection expression of supersite transplants in 96-well microplates

A 96-well microplate-formatted transient gene expression approach was used to achieve high-throughput expression and screening of various scaffold proteins. HEK 293T cells (Invitrogen, CA) were thawed and incubated with growth medium (High Glucose Dulbecco's Modified Eagle Medium with 10% Fetal Bovine Serum and 1% streptomycin-penicillin) (Invitrogen, CA) at 37°C, 5% CO_2_, until the cells reached logarithmic physiological growth. 24 hours prior to DNA-transient transfection, 100 µl of physiologically growing cells was seeded in each well of a 96-well microplate at a density of 2.5×10^5^ cells/ml in expression medium (High Glucose Dulbecco's Modified Eagle Medium supplemented with 10% Ultra-Low IgG Fetal Bovine Serum and 1x-Non-Essential Amino Acids) (Invitrogen, CA), and incubated at 37°C, 5% CO_2_ for 20 hours. Two hours prior to transfection, 100 µl of spent medium from each well was replaced with 60 µl of fresh expression medium.

DNA-TrueFect-Max complexes were used for transfection, and these were prepared by mixing 0.2 µg plasmid DNA in 10 µl of Opti-MEM transfection medium (Invitrogen, CA) with 0.4 µl of TrueFect-Max (United BioSystems, MD) in 10 µl of Opti-MEM, and incubating for 15 min prior to transfection. 20 µl of the complex was added into each well and mixed with growing cells, and the 96-well microplate was incubated at 37°C, 5% CO_2_. One day post transfection, 20 µl of enriched medium (High Glucose Dulbecco's Modified Eagle Medium plus 25% Ultra-Low IgG Fetal Bovine Serum, 2x Non-Essential Amino Acids, 1x glutamine) was added to each well, and returned to incubator for continuous culture. On days three to five post transfection, the culture was exposed to oxygen in the sterilized air once per day. Five-day post transfection, the expressed transplant protein titer in the supernatant in 96-well microplate was quantified using an Octet RED384 (ForteBio, CA). For HEK GnTi- cell transient gene expression, High Glucose Dulbecco's Modified Eagle Medium was replaced with 293 FreeStyle Expression Medium (Invitrogen, CA). Kifunensine (Enzo Life Sciences, NY) at 12.5 mg/L was used in the expression of glycan V3 transplants to ensure the addition of Man_9_ glycans to the recombinant proteins.

### Quantification of supersite transplant expression by Octet assay

The titers of His-tagged supersite transplant proteins in expression supernatants were quantified by Octet RED384. Anti-Penta-His biosensors (ForteBio, CA) were presoaked in 1X Kinetics buffer (ForteBio, CA). 40 µl of supernatant expressed in 96-well microplate was diluted in 160 µl of PBS for analysis, with 40 µl of non-transfection culture medium diluted in 160 µl of PBS as a negative control. Standard curves were generated using serial diluted His-tagged protein with known concentration. All sensorgrams were obtained over 200 seconds at 30°C at a shake speed of 1000 rpm. The titers of supersite transplant proteins were automatically calculated using the Octet analysis software.

### Quantification of supersite transplant expression by ELISA

30 µl of supernatant from each well of the 96-well microplate was mixed with 70 µl of PBS, coated onto Maxisorb (Fisher Scientific, PA) 96-well ELISA plates and incubated overnight at 4°C. The wells were washed once with wash buffer (PBS+0.05% Tween 20), and 300 µl of blocking buffer (PBS with 5% w/v dry milk) was added to each well and incubated for 2 hours at room temperature (RT). The wells were then washed 5 times with wash buffer. 100 µl of mouse Anti-Penta-His primary antibody (QIAGEN, CA) at 0.1 µg/ml was added to each well and incubated for 1 hour at RT and the wells were washed 5 times with wash buffer. Horseradish peroxidase (HRP)-conjugated goat anti-mouse antibody (Jackson ImmunoResearch Laboratories Inc., PA) at 1∶5,000 was added to each well and incubated for 1 hour at RT. The plate was then washed 5 times with wash buffer and developed using 3,3′,5,5′-tetramethylbenzidine (TMB) (Kirkegaard & Perry Laboratories, MD) at RT for 10 min before the reaction was stopped with 180 mM HCl. The readout was measured at a wavelength of 450 nm. All samples were performed in duplicate.

### Binding assay of supersite transplant by ELISA

30 µl of supernatant from each well of the 96-well microplate was mixed with 70 µl of PBS and incubated in each well of a Nickel coated 96-well ELISA plate (Thermo Scientific, IL) for two hours at room temperature (RT) or overnight at 4°C. The well was washed once with wash buffer, and then incubated with 100 µl of respective anti-HIV-1 antibody at a concentration of 10 µg/ml in PBS+0.05% Tween 20+0.2% w/v dry milk for 1 hour at RT. After 5-time wash with wash buffer, the wells were incubated with 100 µl of Horseradish peroxidase (HRP)-conjugated goat anti-human IgG Fc antibody (Jackson ImmunoResearch Laboratories Inc., PA) at 1∶10,000 in PBS+0.05% Tween 20+1% w/v dry milk for 30 min at RT. The wells were then washed 5 times with wash buffer and developed using TMB at RT for 10 min before addition of 180 mM HCl. The readout was measured at a wavelength of 450 nm. All samples were performed in duplicate.

### Expression and purification of antibody IgG and supersite transplant recombinant proteins

For each 1 L expression, 500 µg of supersite transplant plasmid DNA (or 250 µg of each heavy chain and light chain plasmid DNAs) were added to 20 ml of Opti-MEM transfection medium (Invitrogen, CA) and combined with 20 ml of Opti-MEM transfection medium containing 1 ml of TrueFect-Max transfection reagent (United Biosystems, Bethesda, MD). The DNA-TrueFect-Max complexes were incubated for 20 minutes at room temperature before adding to 800 ml of FreeStyle 293F cell culture (1.4×10^6^ cells/ml) in a 2-L shaking flask. For expression of the glycan V3-supersite transplant proteins, FreeStyle 293F cells were pre-incubated with glycan inhibitor kifunensine (Enzo Life Sciences, NY) at 12.5 mg/L or swainsonine at 10 mg/L for 2 hours prior to transfection. The cell culture was returned to suspension incubation for 24 hours at 37°C, 8% CO_2_ and 125 rpm, and then fed with 100 ml of enriched CellBoost-5 medium (HyClone, Logan, UT) containing 1 mM valproic acid (SIGMA, St. Louis, MO) (for antibodies) or sodium butyrate (SIGMA, St. Louis, MO) (for supersite transplant proteins) as expression enhancer. The supernatant was harvested 6 days post transfection by centrifugation and filtered through 0.22 µm filter. The antibody IgG was purified by Protein A affinity column.(Protein A Plus Agarose, Thermo Scientific, Rockford, IL). The supersite transplant proteins were purified by Ni-NTA column (Ni-NTA resin, QIAGEN, Valencia, CA) followed by gel filtration with a Superdex S200 column (GE Healthcare, NJ).

### Surface plasmon resonance

Binding affinities of the supersite transplants and their variants to antibodies were assessed by surface plasmon resonance on a Biacore T-200 (GE Healthcare) at 20°C with buffer HBS-EP^+^ (10 mM HEPES, pH 7.4, 150 mM NaCl, 3 mM EDTA, and 0.05% surfactant P-20). In general, mouse anti-human Fc antibody was first immobilized onto a CM5 chip at 8000-10000 response units (RU) with standard amine coupling protocol (GE Healthcare). Human anti-HIV antibody IgG were then captured with the immobilized anti-human Fc antibody to 250–500 response units (RUs). Site transplants at 2-fold increasing concentrations were injected over the captured antibody channel and a reference channel without captured IgG at a flow rate of 30 µl/min for 3 minutes and allowed to dissociate for another 5 minutes before regeneration with two 25 µl injections of 3.0 M MgCl_2_ at a flow rate of 50 µl/min. For proteins with high non-specific binding to the CM5 chip or mouse anti-human Fc antibody, a 1-minute injection of 2 µM human Fc after the IgG capture step was used to block unliganded mouse anti-human Fc antibody. Blank sensorgrams were obtained by injection of same volume of HBS-EP^+^ buffer in place of site transplant proteins. Sensorgrams of the concentration series were corrected with corresponding blank curves and fitted globally with Biacore Evaluation software (GE Healthcare) using a 1∶1 Langmuir model of binding.

### Structural analysis of glycan V3 supersite

To compare interactions between the glycan V3 supersite and human antibodies that target this site, we aligned the eODmV3:PGT128 (PDB ID 3TYG) and JRFLgp120:PGT135 (PDB ID 4JM2) complexes to the trimeric BG505 SOSIP gp40:PGT122 complex (PDB ID 4NCO) over the gp120 outer domain region. The orientations of approaching antibodies were visualized with PyMol [70] and the gp120:antibody interfaces were analyzed with program PISA [71]. Contributions of peptides and glycans inside or outside the glycan V3 region to the epitope of each antibody were compared.

### Ferritin nanoparticles of glycan V3-supersite transplants

The glycan V3-supersite transplants were placed onto self-assembling *Helicobacter pylori* non-haem ferritin (PDB ID 3BVE) nanoparticles by fusing the supersite transplants to the N-terminus of ferritin with a flexible GGGGSG linker. The fusion proteins were expressed in 293F cells in the presence of kifunensine with protocols described above and purified with Lentil Lectin-Sepharose affinity column (GE Healthcare, NJ).

## Supporting Information

Figure S1 **Flowchart for computational design of supersite transplants.**
(TIF)

Figure S2 **V1V2-supersite transplants bind to antibodies from multiple donors.** All supersite transplants which showed binding to PG9 in the 96 well screen were expressed at 1 liter scale, purified and tested for binding to V1V2-directed antibodies from multiple donors by ELISA. Supersite transplants ST01 (derived from PDB ID 2ZJR) and ST04 (derived from 1VH8) showed weak binding to several antibodies.
(TIF)

Figure S3 **Models of glycan V3-supersite transplants in complex with antibody PGT128.** The 11 glycan V3-supersite transplants with significant PGT128 reactivity were shown with grafted mini-V3 colored red and TM-align identified acceptor scaffolds colored gray and labeled with their PDB IDs. The overlapping red and gray strands indicated the location of transplantation. The glycans at Asn301 and Asn332 were colored cyan in sticks representation. Antibody PGT128 were shown with heavy chain colored blue and light chain colored green.
(TIF)

File S1 **Supplementary Tables.**
(PDF)
