# Correction: Transplanting Supersites of HIV-1 Vulnerability

**DOI:** 10.1371/journal.pone.0105659

**Published:** 2014-08-11

**Authors:** The PLOS ONE Staff

The fifteenth author’s name is spelled incorrectly. The correct name is: Peter D. Kwong.
