# Supplementary figures and images for: Transplanting Supersites of HIV-1 Vulnerability
Source: PLoS One. 2014 Jul 3;9(7):e99881. doi: 10.1371/journal.pone.0099881 (PMC4084637; doi:10.1371/journal.pone.0099881)

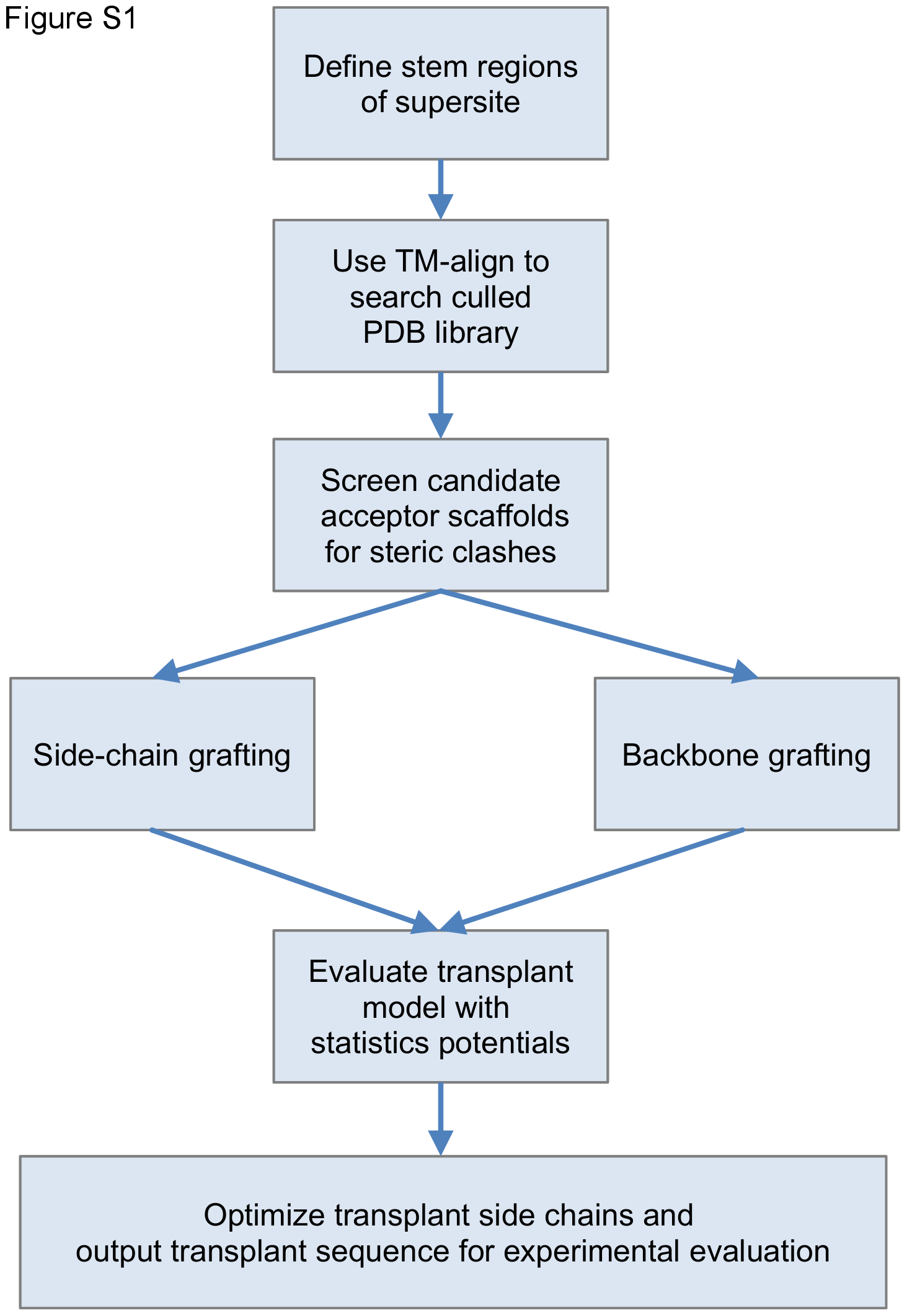

Supplement: Figure S1 — Flowchart for computational design of supersite transplants. (TIF) [file pone.0099881.s001.tif]

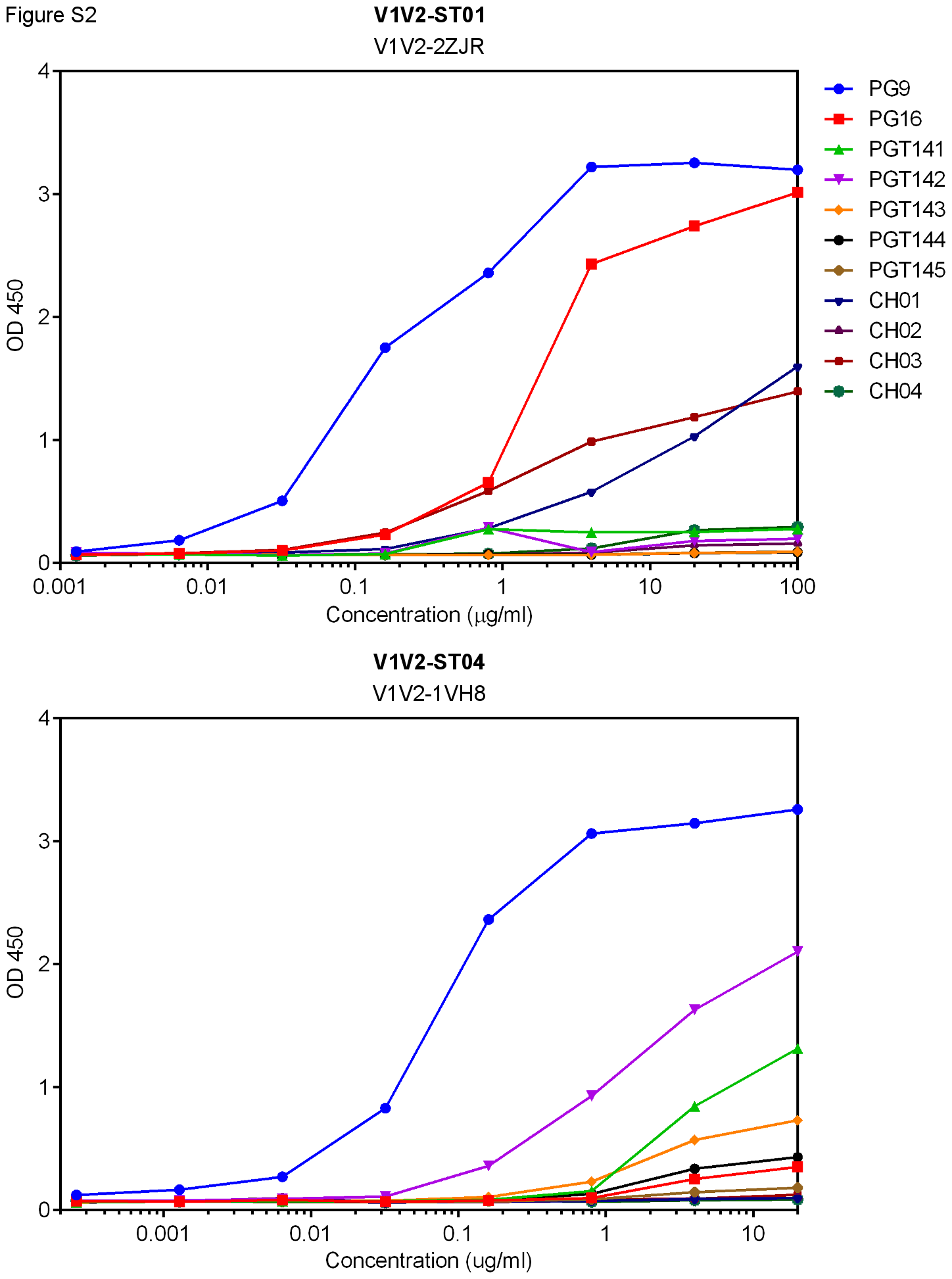

Supplement: Figure S2 — V1V2-supersite transplants bind to antibodies from multiple donors. All supersite transplants which showed binding to PG9 in the 96 well screen were expressed at 1 liter scale, purified and tested for binding to V1V2-directed antibodies from multiple donors by ELISA. Supersite transplants ST01 (derived from PDB ID 2ZJR) and ST04 (derived from 1VH8) showed weak binding to several antibodies. (TIF) [file pone.0099881.s002.tif]

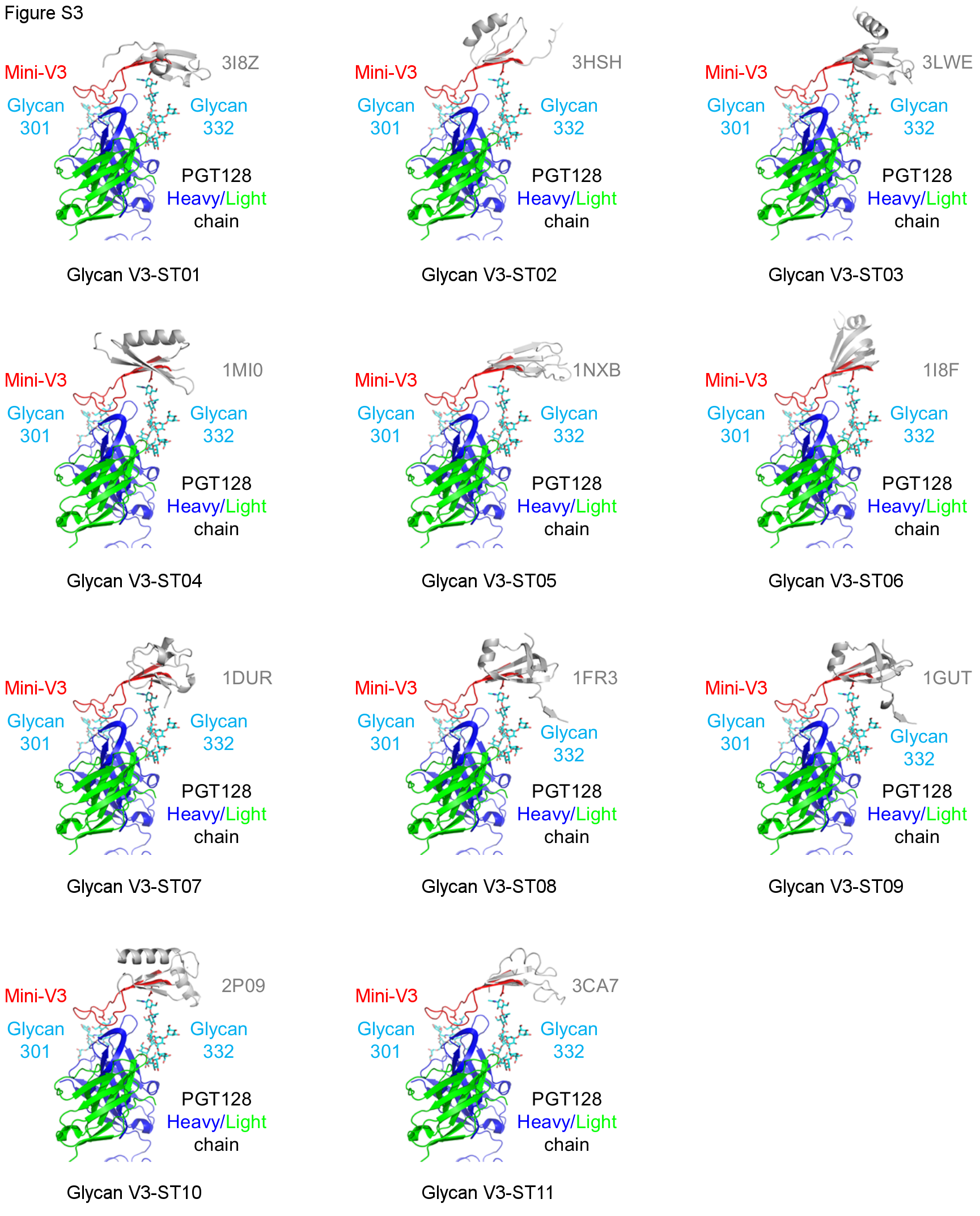

Supplement: Figure S3 — Models of glycan V3-supersite transplants in complex with antibody PGT128. The 11 glycan V3-supersite transplants with significant PGT128 reactivity were shown with grafted mini-V3 colored red and TM-align identified acceptor scaffolds colored gray and labeled with their PDB IDs. The overlapping red and gray strands indicated the location of transplantation. The glycans at Asn301 and Asn332 were colored cyan in sticks representation. Antibody PGT128 were shown with heavy chain colored blue and light chain colored green. (TIF) [file pone.0099881.s003.tif]
