# Supplementary material for: Transplanting Supersites of HIV-1 Vulnerability
Source: PLoS One. 2014 Jul 3;9(7):e99881. doi: 10.1371/journal.pone.0099881 (PMC4084637; doi:10.1371/journal.pone.0099881)
Supplement: File S1 — Supplementary Tables. (PDF) [file pone.0099881.s004.pdf]

Table S1. Design and sequence information of 47 gp41 MPER-supersite transplants.[a]

PDB ID (Chain ID)	MPER-supersite transplant	Epitope transplantation and mutations	Amino-acid sequence
2CW9 (A)		4-32 -> 10E8 epitope, 10 aa grafted, K43A	NAFIRASRALTDKVTSLLNWFFSTNELWEVRTEILRVDPAFDADRFLKQCENDIIPNVLEAMISGELDILKDWCYEATYSQLAHPIQQAKALGLQFHSRILDIDNVDLAMGKMVEQGPVLIITFQAQLVMVVRNPKGEVVEGDPDKVLRMLYVWALCRDQDELNPYAAWRLLDISASSTEQI
2CW9 (A)		4-32 -> 10E8 epitope, 11 aa grafted, K43A	NAFIRASRALTDKVTSLWNWFFSTNELWEVRTEILRVDPAFDADRFLKQCENDIIPNVLEAMISGELDILKDWCYEATYSQLAHPIQQAKALGLQFHSRILDIDNVDLAMGKMVEQGPVLIITFQAQLVMVVRNPKGEVVEGDPDKVLRMLYVWALCRDQDELNPYAAWRLLDISASSTEQI
2CW9 (A)		4-32 -> 10E8 epitope, 12 aa grafted, K43A	NAFIRASRALTDKVTSLWNWFFSTNELWEIRTEILRVDPAFDADRFLKQCENDIIPNVLEAMISGELDILKDWCYEATYSQLAHPIQQAKALGLQFHSRILDIDNVDLAMGKMVEQGPVLIITFQAQLVMVVRNPKGEVVEGDPDKVLRMLYVWALCRDQDELNPYAAWRLLDISASSTEQI
2CW9 (A)		4-32 -> 10E8 epitope, 15 aa grafted, K43A	NAFIEQSLLLTDKVTSLWNWFFSTNELWEIRTEILRVDPAFDADRFLKQCENDIIPNVLEAMISGELDILKDWCYEATYSQLAHPIQQAKALGLQFHSRILDIDNVDLAMGKMVEQGPVLIITFQAQLVMVVRNPKGEVVEGDPDKVLRMLYVWALCRDQDELNPYAAWRLLDISASSTEQI
3C8I (A)	1.1	88-113 -> 10E8 epitope, 10 aa grafted	DLVPAMIAEVNPRDMVVMALVNTNVDPTLPPRWALATRNITAIPGIEGDTRKVGTRIPAVAVTGQRSVGNQDSWDQISPMPIAWATPDSSVIARAESLINWFQWTNLLWNLRKLDQVRETKFDLLEL
3C8I (A)	1.2	88-113 -> 10E8 epitope, 11 aa grafted	DLVPAMIAEVNPRDMVVMALVNTNVDPTLPPRWALATRNITAIPGIEGDTRKVGTRIPAVAVTGQRSVGNQDSWDQISPMPIAWATPDSSVIARAESLWNWFQWTNLLWNLRKLDQVRETKFDLLEL
3C8I (A)	1.3	88-113 -> 10E8 epitope, 12 aa grafted	DLVPAMIAEVNPRDMVVMALVNTNVDPTLPPRWALATRNITAIPGIEGDTRKVGTRIPAVAVTGQRSVGNQDSWDQISPMPIAWATPDSSVIARAESLWNWFQWTNLLWNIRKLDQVRETKFDLLEL
3C8I (A)	1.4	88-113 -> 10E8 epitope, 13 aa grafted	DLVPAMIAEVNPRDMVVMALVNTNVDPTLPPRWALATRNITAIPGIEGDTRKVGTRIPAVAVTGQRSVGNQDSWDQISPMPIAWATPDSSVIDKAESLWNWFQWTNLLWNIRKLDQVRETKFDLLEL
3C8I (A)	1.5	88-113 -> 10E8 epitope, 15 aa grafted	DLVPAMIAEVNPRDMVVMALVNTNVDPTLPPRWALATRNITAIPGIEGDTRKVGTRIPAVAVTGQRSVGNQDSWDQISPMPIAWATPDLLVIDKAESLWNWFQWTNLLWNIRKLDQVRETKFDLLEL
2UXE (A)		23-48 -> 10E8 epitope, 10 aa grafted	MRTLLIRYILWRNDNDQTYYNDDFKKLMLLDSLVNWFDVTNLLWNMRMTLSDGPLLDRLNQPVNNIEDAKRMIAISAKVARDIGERSEIRWEESFTILFRMIETYFDDLMIDLYG
2UXE (A)		23-48 -> 10E8 epitope, 11 aa grafted	MRTLLIRYILWRNDNDQTYYNDDFKKLMLLDSLWNWFDVTNLLWNMRMTLSDGPLLDRLNQPVNNIEDAKRMIAISAKVARDIGERSEIRWEESFTILFRMIETYFDDLMIDLYG
2UXE (A)		23-48 -> 10E8 epitope, 12 aa grafted	MRTLLIRYILWRNDNDQTYYNDDFKKLMLLDSLWNWFDVTNLLWNIRMTLSDGPLLDRLNQPVNNIEDAKRMIAISAKVARDIGERSEIRWEESFTILFRMIETYFDDLMIDLYG

2UXE (A)		23-48 -> 10E8 epitope, 13 aa grafted	MRTLLIRYILWRNDNDQTYYNDDFKKLDKLDSLWNWFDVTNLLWNIRMTLSDGPL LDRLNQPVNNIEDAKRMIAISAKVARDIGERSEIRWEESFTILFRMIETYFDDLMIDLY G
2UXE (A)		23-48 -> 10E8 epitope, 15 aa grafted	MRTLLIRYILWRNDNDQTYYNDDLLKLDKLDSLWNWFDVTNLLWNIRMTLSDGPLL DRLNQPVNNIEDAKRMIAISAKVARDIGERSEIRWEESFTILFRMIETYFDDLMIDLY G
1ONQ (A)		131-158 -> 10E8 epitope, 10 aa grafted	PLSFHVIWIASFYNHSWKQNLVSGWLSDLQTHTWDSNSSTIVFLWPWSRGNFSN EEWKELETLFRIRTIRSFEGIRRYAHELQFEYPFEIQVTGGCELHSGKVSGSFLQLA YQGSDFVSFQNNSWLPYPVAGNMAKHFCSLLNWFENTNTLWLLRDTCPRFILGLL DAGKAHLQR
1ONQ (A)		131-158 -> 10E8 epitope, 11 aa grafted	PLSFHVIWIASFYNHSWKQNLVSGWLSDLQTHTWDSNSSTIVFLWPWSRGNFSN EEWKELETLFRIRTIRSFEGIRRYAHELQFEYPFEIQVTGGCELHSGKVSGSFLQLA YQGSDFVSFQNNSWLPYPVAGNMAKHFCSLWNWFENTNTLWLLRDTCPRFILGL LDAGKAHLQR
1ONQ (A)		131-158 -> 10E8 epitope, 12 aa grafted	PLSFHVIWIASFYNHSWKQNLVSGWLSDLQTHTWDSNSSTIVFLWPWSRGNFSN EEWKELETLFRIRTIRSFEGIRRYAHELQFEYPFEIQVTGGCELHSGKVSGSFLQLA YQGSDFVSFQNNSWLPYPVAGNMAKHFCSLWNWFENTNTLWLIRDTCPRFILGLL DAGKAHLQR
1ONQ (A)		131-158 -> 10E8 epitope, 13 aa grafted	PLSFHVIWIASFYNHSWKQNLVSGWLSDLQTHTWDSNSSTIVFLWPWSRGNFSN EEWKELETLFRIRTIRSFEGIRRYAHELQFEYPFEIQVTGGCELHSGKVSGSFLQLA YQGSDFVSFQNNSWLPYPVAGNMADKFCSLWNWFENTNTLWLIRDTCPRFILGLL DAGKAHLQR
1ONQ (A)		131-158 -> 10E8 epitope, 15 aa grafted	PLSFHVIWIASFYNHSWKQNLVSGWLSDLQTHTWDSNSSTIVFLWPWSRGNFSN EEWKELETLFRIRTIRSFEGIRRYAHELQFEYPFEIQVTGGCELHSGKVSGSFLQLA YQGSDFVSFQNNSWLPYPVALLMADKFCSLWNWFENTNTLWLIRDTCPRFILGLL DAGKAHLQR
3MHS (B)	2.1	58-83 -> 10E8 epitope, 10 aa grafted	TAQLKSQIQQYLVESGNYELISNELKARLLQEGWVDKVKDLTKSEMNINESTNFTQI LSTVEPKALSLVNWFTRTNVLWQIREFLEEIVDT
3MHS (B)	2.2	58-83 -> 10E8 epitope, 11 aa grafted	TAQLKSQIQQYLVESGNYELISNELKARLLQEGWVDKVKDLTKSEMNINESTNFTQI LSTVEPKALSLWNWFTRTNVLWQIREFLEEIVDT
3MHS (B)	2.3	58-83 -> 10E8 epitope, 13 aa grafted	TAQLKSQIQQYLVESGNYELISNELKARLLQEGWVDKVKDLTKSEMNINESTNFTQI LSTVEDKALSLWNWFTRTNVLWQIREFLEEIVDT
3MHS (B)	2.4	58-83 -> 10E8 epitope, 15 aa grafted	TAQLKSQIQQYLVESGNYELISNELKARLLQEGWVDKVKDLTKSEMNINESTNFTQI LLLVEDKALSLWNWFTRTNVLWQIREFLEEIVDT
1OCK(A)		300-325 -> 10E8 epitope, 10 aa grafted	DLKEAVHEAWRIHPIIQDFEAHAALAWEFAEHHDEIAPMLRASLDATSLLNWFEYT NALWIGRRGRRELGEVFEG
1OCK(A)		300-325 -> 10E8 epitope, 11 aa grafted	DLKEAVHEAWRIHPIIQDFEAHAALAWEFAEHHDEIAPMLRASLDATSLWNWFEYT NALWIGRRGRRELGEVFEG
1OCK(A)		300-325 -> 10E8 epitope, 12 aa grafted	DLKEAVHEAWRIHPIIQDFEAHAALAWEFAEHHDEIAPMLRASLDATSLWNWFEYT NALWIIRRGRRELGEVFEG
1OCK(A)		300-325 -> 10E8 epitope, 13 aa grafted	DLKEAVHEAWRIHPIIQDFEAHAALAWEFAEHHDEIAPMLRASLKATSLWNWFEYT NALWIIRRGRRELGEVFEG
3AGJ (A)	3	Y26A, K52G, E49G, E33W, K34F, K37T, E38N, E40L, E41W, K44R, S45A, delete(cterm 128aa)	KPHMNLVVIGHVDHGKSTLVGHLLARLGYIEWFKLTNLLWQARARGKGSFGFAWI LDKMKEERERGITIDLTFMKFETKKYVFTIIDAPGHRDFVKNMITGASQADAAILVVS ARKGEFEAGMSTEGQTREHLLLARTMGIEQIIVAVNKMDAPDVNYDQKRYEFVVS

		VLKKFMKGLGYQVDKIPFIPVSAWKGDNLIERSPNMPWYNGPTLVEALDQLQPPAK
2NN4 (A)	L32A, E45N, K46W, E47F, W49I, A50T, R51N, A53L, A54W	LNTFYDVQQLLKTFGHIVYFGDRELEIEFMADELKELYMNHMINWFQITNALWVLRKELEQT
2NN4 (A)	Y39A, L32A, E45N, K46W, E47F, W49I, A50T, R51N, A53L, A54W	LNTFYDVQQLLKTFGHIVYFGDRELEIEFMADELKELAMNHMINWFQITNALWVLRKELEQT
3DMA (A)	K4S, R155A, Y158A, A7N, Q8W, A9F, D12T, H13N, T15L, K16W, E19R	LTSVINWFHITNFLWWFRRADKIVIVSHVSPDGDAIGSSLGLYHFLDSQDKIVNVIVPNAFPDFLKWMPGSKDILLYDRYQEFADKLIMEADVICCLDFNALKRIDEMSDIVAASPGRKIMIDHHLYPEDFCRITISHPEISSTSELVFRLICAMGAFSDISKEGAECIYTGMMTDTGGFTYNSNNREIYFIISELLSKGIDKDDIYRKVYNT
3KZ5 (A)	T31W, E32F, E35T, E38L, A39W, K42R	SSRHQFAPGATVLYKGDKMVLNLDRSRVPWFCITKILWILRELEKPA
3KZ5 (A)	T31W, E32F, E35T, K36N, E38L, A39W, K42R, E45A	SSRHQFAPGATVLYKGDKMVLNLDRSRVPWFCITNILWILRELAKPA
2QRW (A)	D84S, L87A, H91A, E100A, delete(T101), D104W, D105F, R107I, R108T, D113W, E116R	KSFYDAVGGAKTFDAIVSRFYAQVAEDEVLRRVYPEDDLAGAEERLRMFLEQYWGGPRTYSEQRGHPRLRMRHAPFRISLIERSAFARCMATAVASIDSALDWFHITELLWYLRMAAHSLVNSPF
3GJ0 (A)	V189G, P192W, A193F, A196T, Q197N, E199L, H200W, E203R	QVQFKLVLVGDGGTGKTTFVKRHLTGEFEKKYVATLGVEVHPLVFHTNRGPIKFNVWDTAGQEKFGGLRDGYYIQAQCAIIMFDVTSRVTYKNVPNWHRDLVRVCENIPIVLCGNKVDIKDRKVKAKSIVFHRKKNLQYYDISAKSNYNFEKPFLWLARKLIGDPNLEFVAMPALAPPEVGMDWFLATNYLWDLRVAQTT
3KVH (A)	F168A, K171A, N174A, E178W, E179F, V182T, E183N, A186W, T189R	GWSHSCHAMLYAANPGQLFGRIPMRFSVLMQMRFDGLLGFPGGFVDRRFWSLEDGLNRVLGLGLGSGRLTEADYLSSHLTGSGRVVAHLYARQLTLEQLHAVEISAVHSRDHGLEVLGLVRVPLYTQKDRVGGFPNFLSNAFVSTAKCQLLFALKVLNMMPEEKLVEALAAATEKQKKALEKLL
3IX3 (A)	L11G, E42G, N49L, A52W, A53F, R55I, E56T, H57N, D59L, R60W	FLELERSSGKGEWSAILQKMASDLGFSKILFGLLPKDSQDYGNAFIVGLYPWFWITNYLWAGYARVDPTVSHCTQSVLPIFWEPSIYQTRKQHEFFEEASAAGLVYGLTMPLHGARGELGALSLSVEAENRAEANRFMESVLPTLWMLKDYALQSGAGLAFEH
1UB4 (A)	insert(18<-SGS->26), T93G, P96W, E97F, Q100T, K103L, A104W, N107R	VSRYVPDMGDLIWVDFDPSGSGHRPAVVLSPFMYNDKTGMCLCVPCTTQSKGYPFEVVLSGQERDGVALADQVKSIAWRARGATKKGGVAWFELTLILWKIRVLIG
1UB4 (A)	insert(18<-SGS->26), T93G, P96W, E97F, Q100T, L101N, K103L, A104W, N107R	VSRYVPDMGDLIWVDFDPSGSGHRPAVVLSPFMYNDKTGMCLCVPCTTQSKGYPFEVVLSGQERDGVALADQVKSIAWRARGATKKGGVAWFELTNILWKIRVLIG
1U6Z (A)	delete (Nterm 300aa), N314S, H317A, E350A, R354A, S320W, E321F, R324T, R325N, D328W, M331R	QDVRSRTASSLASQYAIDWFQATNVLWTTRQMYEQWREQQPKLAHPQLAALLAWAAMLHEVGLNINHAGLHRHSAYILQNSDLPGFNQEQQLMMATLVRYHRKAIKLDDLPRFTLFKKKQFLPLIQLLRLGVLLNNQRQATTTPPTLTLITDDSHWTLRFPHDWFSQNALVLLDLEKEQEYWEGVAGWRLKIEEESTP
2CJW (A)	K175W, E176F, E179T, R180N, A183W, K186R	MTYYRVVLIGEQGVGKSTLANIFAGVHDSGSGSCEVLGEDTYERTLMVDGESATIILLDMWEDGSGSEWLHDHCMQVGDAYLIVYSITDRASFEKASELRIQLRRARQTEDIPIILVGNKADLVRCREVSVSEGRACAVVFDCKFIETSAAVQHNVKELFEGIVRQVRLRRDSWFKNTNRLWYQRR
2D4U (A)	E90A, R94A, P96S, T100W, A101F, A104T, E105N, K107L, R108W, D111R	GPLGSGGLFFNALKNCKENFAVLQTIRQQQSTLNGAWVALLQTRNTLNRAGIRYMMDQNNIGSGSTVAELMESASISLKQAEKNWADYAALPADSRQSWFAATNILWNYR

		IYHNALAELIQLLGAGKINEFFDQPTQGYQDGFEKQYVAYMEQNDRLHDIAVSDNNA
3AG3.1 (H)	I60W, S61F, S64T, D67L, D68W, R70I, A71R	KIKNYQTAPFDSRFPNQNQARNCWQNYLDFHRCEKAMTAKGGDVSVCEWYRRVYKSLCPWFWVTTWLWRIREGTFPGKI
3AG3.2 (H)	K55A, I60W, S61F, S64T, T65N, D67L, D68W, R70I, A71R	KIKNYQTAPFDSRFPNQNQARNCWQNYLDFHRCEKAMTAKGGDVSVCEWYRRVYASLCPWFWVTNWLWRIREGTFPGKI
3HW5 (A)	R10A, P15W, M16F, E19T, L20N, E22L, K23W, K26R, insert(54<-GSGSG->80)	PLGSMEDFVAQCFNWFIVTNALWAMREYGEDPKIETNKFAAICTHLEVCFMYSGSGSGRFEIIEGRDRTMAWTVVNSICQTTGVEKPKFLPDLYDYKENRFIEIGVTRREVHTYYLEKANKIKSEKTHIHIFSFTGEEMATKADYTLDEESRARIKTRLFTIRQEMASRGLWDSFRQSERGE
3IUO.1 (A)	E61S, E65W, D66F, E69T, E73W, K76R	RVRTLANKAKMKVSIVQQIDRKVALDDIAVSHGLDFPELLSEVETIVYSGTRINIDYFINSVMDWFHLTDIFWYFRESTTDSLEEAMQELGKDYSEEEIRLVRIKFLSEM
3IUO.2 (A)	E61A, E65W, D66F, E69T, D70N, F72L, E73W, K76R	RVRTLANKAKMKVSIVQQIDRKVALDDIAVSHGLDFPELLSEVETIVYSGTRINIDYFINAVMDWFHLTNILWYFRESTTDSLEEAMQELGKDYSEEEIRLVRIKFLSEM

[a] Listed items include epitope-scaffold name, PDB identifier, brief description of epitope transplantation and mutations made in the computational design, and amino acid sequence of the epitope-scaffold. Scaffolds 10E8-ES1 through 10E8-ES6 and 10E8-ES15 through 10E8-ES21were appended with C-terminal Hisx8/Strep tags. Scaffolds 10E8-ES7 through 10E8-ES14 were appended with C-terminal eGFP/Hisx8 tags in an attempt to improve solubility.

Table S2. Design and sequence information of 67 PG9-epitope scaffolds.[a]

PDB ID (Chain ID)	V1V2-supersite transplant	Epitope transplantation and mutations	Amino-acid sequence
2JNI (A)	-	Y7N + R9T	RWCVYANVTIRGVLVRYRRCW
2JNI (A)	-	Y7N + R9T, C3F + R18N + C20T, insert (V-Nterm and Cterm-Y)	VCWFVYANVTIRGVLVRYNRTCY
3BW1 (A)	-	46-67 -> 154-177	HHMETPLDLLKLNLDERVYIKLRGARTLVGTLQAFDSHCNIVLSVKH CSFNITTDVKDRKQKVNATFYMVFIRGDTVTLISTPS
3BW1 (A)	-	46-67 -> 154-177, V154D, C157A, Y177E	HHMETPLDLLKLNLDERVYIKLRGARTLVGTLQAFDSHCNIVLSDKH ASFNITTDVKDRKQKVNATFEMVFIRGDTVTLISTPS
3BW1 (A)	-	46-67 -> 154-177, V154D, C157A, Y177E, S45C + M68C	HHMETPLDLLKLNLDERVYIKLRGARTLVGTLQAFDSHCNIVLCDKH ASFNITTDVKDRKQKVNATFECVFIRGDTVTLISTPS
2QLD (A)	-	delete 85-174, 21-44 -> 154-177	PPVTHDLRVSLEEIYSGCTKVKHCSFNITTDVKDRKQKVNATFYIEV KKGWKEGTKITFPKEGDQTIPADIVFVLKDKPHN
2QLD (A)	-	delete 85-174, 21-44 -> 154-177, L11T, C157A	PPVTHDLRVSTEEIYSGCTKVKHASFNITTDVKDRKQKVNATFYIEV KKGWKEGTKITFPKEGDQTIPADIVFVLKDKPHN
2QLD (A)	-	delete 85-174, 21-44 -> 154-177, L11T, C157A, F159H, I161R	PPVTHDLRVSTEEIYSGCTKVKHASHNRTTDVKDRKQKVNATFYIE VKKGWKEGTKITFPKEGDQTIPADIVFVLKDKPHN
2ZJR (Q)	1.1	55-78 -> 154-177, K31G, Y177A	SHYDILQAPVISEKAYSAMERGVYSFWVSPGATKTEIKDAIQQAFG VRVIGISVKHCSFNITTDVKDRKQKVNATFAIVRLAEGQSIEALAGQ
2ZJR (Q)	1.2	55-78 -> 154-177, K31G, V154T, Y177A, C157A	SHYDILQAPVISEKAYSAMERGVYSFWVSPGATKTEIKDAIQQAFG VRVIGISTKHASFNITTDVKDRKQKVNATFAIVRLAEGQSIEALAGQ
2BKY (A)	-	62-84 -> 154-177	SNVVLIGKKPVMNYVLAALTLLNQGVSEIVIKARGRAISKAVDTVEIV RNRFLPDKIEIKEVKHCSFNITTDVKDRKQKVNATFYAIRKK
2BKY (A)	-	62-84 -> 154-177, Y177I, C157A	SNVVLIGKKPVMNYVLAALTLLNQGVSEIVIKARGRAISKAVDTVEIV RNRFLPDKIEIKEVKHASFNITTDVKDRKQKVNATFIAIRKK

2VQE (Q)	-	delete 80-104, 19-42 -> 154-177, K155V	PKKVLTGVVVSDKMQKTVVVHCSFNITTDVKDRKQKVNATFYAHD PEEKYKLGDVVEIIESRPISKRKRFRVLRLVESG
2VQE (Q)	-	delete 80-104, 19-42 -> 154-177, K155V, C157A	PKKVLTGVVVSDKMQKTVVVHASFNITTDVKDRKQKVNATFYAHD PEEKYKLGDVVEIIESRPISKRKRFRVLRLVESG
2VQE (Q)	-	delete 80-104, 19-42 -> 154-177, K155V, C157A, F159R	PKKVLTGVVVSDKMQKTVVVHASRNITTDVKDRKQKVNATFYAHD PEEKYKLGDVVEIIESRPISKRKRFRVLRLVESG
1APY (B)	-	121-142 -> 156-177, C157F, F176C, Y177I	TIGMVVIHKTGHIAAGTSTNGIKFKIHGRVGDSPIPGAGAYADDTAG AAAATGNGDILMRFLPSYQAVEYMRRGEDPTIACQKVISRIQKHFP EFFGAVICANVTGSYGAACNKLSTFTHFSFNITTDVKDRKQKVNAT CI
3DDC (B)	2	37-85 -> 154-177, K155V, C157L, F159L, I161R	PPTIQEIKQKIDSYNSREKHCLGMKLSEDGTYTGFIVVHLSLNRTTD VKDRKQKVNATFYMHISSTTTVSEVIQGLLDKFMVVDNPQKFALFK RIHKDGQVLFQKLSIADYPLYLRLLAGPDTDVLSFVLKEN
3HRD (D)	-	1-20 -> 154-175, V154M, C157I	MKHISFNITTDVKDRKQKVNATPNKRLLDLLREDFGLTSVKEGCSE GECGACTVIFNGDPVTTCCMLAGQADESTIITLEGVAEDGKPSLLQ QCFLEAGAVQCGYCTPGMILTAKALLDKNPDPTDEEITVAMSGNLC RCTGYIKIHAAVRYAVERCANAAA
3HRD (D)	-	1-20 -> 154-175, V154M, C157I, Q170R, V172I, A174T	MKHISFNITTDVKDRKRKINTTPNKRLLDLLREDFGLTSVKEGCSEG ECGACTVIFNGDPVTTCCMLAGQADESTIITLEGVAEDGKPSLLQQ CFLEAGAVQCGYCTPGMILTAKALLDKNPDPTDEEITVAMSGNLCR CTGYIKIHAAVRYAVERCANAAA
1YN3 (A)	-	1-32 -> 154-177, V154G, K155S, C157V	GSHVSFNITTDVKDRKQKVNATFYKNQNISYKDLEGKVKSVLESNR GITDVDLRLSKQAKYTVNFKNGTKKVIDLKSGIYTANLINSSDIKSINI NID
1WOC (A)	-	32-49 -> 154-177, K155H	TNRLVLSGTVCRAPLRKVSPSGIPHCQFVLVHHCSFNITTDVKDRK QKVNATFYMPVIVSGHENQAITHSITVGSRITVQGFISCHKAKNGLS KMVLHAEQIELI
1WOC (A)	-	32-49 -> 154-177, K155H, C157A	TNRLVLSGTVCRAPLRKVSPSGIPHCQFVLVHHASFNITTDVKDRK QKVNATFYMPVIVSGHENQAITHSITVGSRITVQGFISCHKAKNGLS KMVLHAEQIELI

1WOC (A)	-	32-49 -> 154-177, K155H, C157A, L31C + M50C	TNRLVLSGTVCRAPLRKVSPSGIPHCQFVCVHHASFNITTDVKDRK QKVNATFYCPVIVSGHENQAITHSITVGSRITVQGFISCHKAKNGLS KMVLHAEQIELI
2ZPM (A)	-	47-66 -> 155-176, C157L, F150L, F176P	PVLENVQPNSAASKAGLQAGDRIVKVDGQPLTQWVTFVMLVRDNP GKHLSLNITTDVKDRKQKVNATPESKPGNGKAIGFVGIEPKVI
1LFD (A)	-	1-26 -> 154-177, V154G, K155D, F159I, I161V, F176S, K39A, N41A	GDHCSINVTTDVKDRKQKVNATSYDKAPTVIRKAMDAHALDEDEPE DYELLQIISEDHKLKIPENANVFYAMNSAANYDFILKKR
1T3Q (A)	-	1-23 -> 154-177, V154S, C157M, F176P, Y177R	SKHMSFNITTDVKDRKQKVNATPRMHLADALREVVGLTGTKIGCEQ GVCGSCTILIDGAPMRSCLTLAVQAEGCSIETVEGLSQGEKLNALQ DSFRRHHALQCGFCTAGMLATARSILAENPAPSRDEVREVMSGNL CRCTGYETIIDAITDPAVAEAARRGEV
2IAB (A)	-	24-43 -> 156-175, C157A	TTPPARTAKQRIQDTLNRLELDVHASFNITTDVKDRKQKVNATYLW DGETFLVATPAASPTGRNLSETGRVRLGIGPTRDLVLVEGTALPLE PAGLPDGVGDTFAEKTGFDPRRLTTSYLYFRISPRRVQAWREANE LSGRELMRDGEWLVTD
3NEC (A)	-	49-67 -> 157-175, C157H	SDWDPVVKEWLVDTGYCCAGGIANAEDGVVFAAAADDDDGWSKL YKDDHSFNITTDVKDRKQKVNATEASTIKAAVDDGSAPNGVWIGG QKYKVVRPEKGFEYNDCTFDITMCARSKGGAHLIKTPNGSIVIALYD EEKEQDKGNSRTSALAFAEYLHQSGY
2VXS (A)	3	58-86 -> 157-175, C157I, F159Q	TNPKRSSDYYNRSTSPWNLHRNEDPERYPSVIWEAKCRHLGCINA DGNVDYHMNSISQNITTDVKDRKQKVNATCTCVTPIVH
1NF3 (C)	-	44-65 -> 154-177, V154I, K155R, C157G, F159S, I161R, Y177I	IVISMPQDFRPVSSIIDVDILPETHRRVRLCKYGTEKPLGFYIRHGSS NRTTDVKDRKQKVNATFISRLVPGGLAQSTGLLAVNDEVLEVNGIE VSGKSLDQVTDMMIANSRNLIITVRPANQRN
2HQL (A)	-	delete 100-104, 28-41 -> 154-177, V154K	MLNRVFLEGEIESSCWSVKKTGFLVTIKKHCSFNITTDVKDRKQKV NATFYYVIYANGQLAYELEKHTKKYKTISIEGILRTYLERKSEIWKTTI EIVKIFNPKNEIVI
2HQL (A)	-	delete 100-104, 28-41 -> 154-177, V154K, C157A	MLNRVFLEGEIESSCWSVKKTGFLVTIKKHASFNITTDVKDRKQKVN ATFYYVIYANGQLAYELEKHTKKYKTISIEGILRTYLERKSEIWKTTIEI VKIFNPKNEIVI

2HQL (A)	-	delete 100-104, 28-41 -> 154-177, V154K, C157A, C15T, I27C + Y42C	MLNRVFLEGEIESSTWSVKKTGFLVTCKKHASFNITTDVKDRKQKV NATFYCVIYANGQLAYELEKHTKKYKTISIEGILRTYLERKSEIWKTTI EIVKIFNPKNEIVI
3FEV (A)	-	5-14 -> 154-177, V154T	LTCVTKHCSFNITTDVKDRKQKVNATFYENCPDGQNLCFKRWQYIS PRMYDFTRGCAATCPKAEYRDVINCCGTDKCNK
3FEV (A)	-	5-14 -> 154-177, V154T, C157A	LTCVTKHASFNITTDVKDRKQKVNATFYENCPDGQNLCFKRWQYIS PRMYDFTRGCAATCPKAEYRDVINCCGTDKCNK
3FEV (A)	-	5-14 -> 154-177, V154T, C157A, K155C + F176C	LTCVTCHASFNITTDVKDRKQKVNATCYENCPDGQNLCFKRWQYI SPRMYDFTRGCAATCPKAEYRDVINCCGTDKCNK
1GVP (A)	-	28-50 -> 154-177, V154L, C157Q, A174I, F176L, Y177D	MIKVEIKPSQAQFTTRSGVSRQGKPYSLKHQSFNITTDVKDRKQKV NATLDEGQPAYAPGLYTVHLSSFKVGQFGSLMIDRLRLVPAK
3EN2 (A)	-	34-47 -> 154-177, insert(H81 + GSG + A86)	AINRLQLVATLVEREVMRYTPAGVPIVNCLLSYVKHCSFNITTDVKD RKQKVNATFYFSIEALGAGKMASVLDRIAPGTVLECVGFLARKHGS GALVFHISGLEHH
3EN2 (A)	-	34-47 -> 154-177, insert (H81 + GSG + A86), C157A	AINRLQLVATLVEREVMRYTPAGVPIVNCLLSYVKHASFNITTDVKD RKQKVNATFYFSIEALGAGKMASVLDRIAPGTVLECVGFLARKHGS GALVFHISGLEHH
3EN2 (A)	-	34-47 -> 154-177, insert(H81 + GSG + A86), C157A, Y33C+F48C	AINRLQLVATLVEREVMRYTPAGVPIVNCLLSCVKHASFNITTDVKD RKQKVNATFYCSIEALGAGKMASVLDRIAPGTVLECVGFLARKHGS GALVFHISGLEHH
1GG3 (A)	-	delete1-185,238-258 -> 156-175, C157F, F159I, D197G, L198G, E199G	SMYGVDLHKAKDLEGVDIILGVCSSGLLVYKDKLRINRFPWPKVLKI SYKRSHFSINITTDVKDRKQKVNATLPSYRAAKKLWKVCVEHHTFF R
2AR5 (A)	-	delete 115-118, 24-44 -> 156-175, C157Y	MDGRIKEVSVFTYHKKYNPDKHYHYSFNITTDVKDRKQKVNATFDE FQELHNKLSIIFPLWKLPGFPNRMVLGRTHIKDVAAKRKIELNSYLQ SLMNASTDVAECDLVCTFFH
2F7S (A)	-	42-69 -> 154-177	DYDYLIKLLALGDSGVGKTTFLYRYTDNKFNPKFITTVGIDVKHCSF NITTDVKDRKQKVNATFYDTAGQERFRSLTTAFFRDAMGFLLMFDL TSQQSFLNVRNWMSQLQANAYCENPDIVLIGNKADLPDQREVNER QARELADKYGIPYFETSAATGQNVEKAVETLLDLIMKRMEQCVE

2F7S (A)	-	42-69 -> 154-177, C157A	DYDYLIKLLALGDSGVGKTTFLYRYTDNKFNPKFITTVGIDVKHASF NITTDVKDRKQKVNATFYDTAGQERFRSLTTAFFRDAMGFLLMFDL TSQQSFLNVRNWMSQLQANAYCENPDIVLIGNKADLPDQREVNER QARELADKYGIPYFETSAATGQNVEKAVETLLDLIMKRMEQCVE
2F7S (A)	-	42-69 -> 154-177, C157A, D41C + D70C	DYDYLIKLLALGDSGVGKTTFLYRYTDNKFNPKFITTVGICVKHASF NITTDVKDRKQKVNATFYCTAGQERFRSLTTAFFRDAMGFLLMFDL TSQQSFLNVRNWMSQLQANAYCENPDIVLIGNKADLPDQREVNER QARELADKYGIPYFETSAATGQNVEKAVETLLDLIMKRMEQCVE
3HM2 (A)	-	149-162 -> 154-177,K155H	GQLTKQHVRALAISALAPKPHETLWDIGGGSGSIAIEWLRSTPQTTA VCFEISEERRERILSNAINLGVSDRIAVQQGAPRAFDDVPDNPDVIFI GGLTAPGVFAAAWKRLPVGGRLVANAVTVESEQMLWALRKQFGG TISSFAIVHHCSFNITTDVKDRKQKVNATFYALPVHQWTVVK
3HM2 (A)	-	149-162 -> 154-177, K155H, C157A	GQLTKQHVRALAISALAPKPHETLWDIGGGSGSIAIEWLRSTPQTTA VCFEISEERRERILSNAINLGVSDRIAVQQGAPRAFDDVPDNPDVIFI GGLTAPGVFAAAWKRLPVGGRLVANAVTVESEQMLWALRKQFGG TISSFAIVHHASFNITTDVKDRKQKVNATFYALPVHQWTVVK
3HM2 (A)	-	149-162 -> 154-177, K155H, C157A, I148C + A163C	GQLTKQHVRALAISALAPKPHETLWDIGGGSGSIAIEWLRSTPQTTA VCFEISEERRERILSNAINLGVSDRIAVQQGAPRAFDDVPDNPDVIFI GGLTAPGVFAAAWKRLPVGGRLVANAVTVESEQMLWALRKQFGG TISSFACVHHASFNITTDVKDRKQKVNATFYCLPVHQWTVVK
1D3B (B)	-	45-57 -> 154-177	SKMLQHIDYRMRCILQDGRIFIGTFKAFDKHMNLILCDCDEFRVKHC SFNITTDVKDRKQKVNATFYEKRVLGLVLLRGENLVSMTVEGPPP
1D3B (B)	-	45-57 -> 154-177, C157A	SKMLQHIDYRMRCILQDGRIFIGTFKAFDKHMNLILCDCDEFRVKHA SFNITTDVKDRKQKVNATFYEKRVLGLVLLRGENLVSMTVEGPPP
1D3B (B)	-	45-57 -> 154-177, C157A, R44C + E58C	SKMLQHIDYRMRCILQDGRIFIGTFKAFDKHMNLILCDCDEFCVKHA SFNITTDVKDRKQKVNATFYCKRVLGLVLLRGENLVSMTVEGPPP
1L3I (A)	-	163-176 -> 154-177	MIPDDEFIKNPSVPGPTAMEVRCLIMCLAEPGKNDVAVDVGCGTG GVTLELAGRVRRVYAIDRNPEAISTTEMNLQRHGLGDNVTLMEGD APEALCKIPDIDIAVVGGSGGELQEILRIIKDKLKPGGRIIVTAILLETK FEAMECLRDLGFDVNITELNIVKHCSFNITTDVKDRKQKVNATFYRN PVALIYTGV

1L3I (A)	-	163-176 -> 154-177, C157A	MIPDDEFIKNPSVPGPTAMEVRCLIMCLAEPGKNDVAVDVGCGTG GVTLELAGRVRRVYAIDRNPEAISTTEMNLQRHGLGDNVTLMEGD APEALCKIPDIDIAVVGGSGGELQEILRIIKDKLKPGGRIIVTAILLETK FEAMECLRDLGFDVNITELNIVKHASFNITTDVKDRKQKVNATFYRN PVALIYTGV
1L3I (A)	-	163-176 -> 154-177, C157A, I162C + R177C	MIPDDEFIKNPSVPGPTAMEVRCLIMCLAEPGKNDVAVDVGCGTG GVTLELAGRVRRVYAIDRNPEAISTTEMNLQRHGLGDNVTLMEGD APEALCKIPDIDIAVVGGSGGELQEILRIIKDKLKPGGRIIVTAILLETK FEAMECLRDLGFDVNITELNCVKHASFNITTDVKDRKQKVNATFYC NPVALIYTGV
1VH8 (A)	4.1	15-32 -> 154-177	SLIRIGHGFDVHAFVKHCSFNITTDVKDRKQKVNATFYFIAHSDGDV ALHALTDAILGAAALGDIGKLFPKNADSRGLLREAFRQVQEKGYKIG NVDITIIAQAPKMRPHIDAMRAKIAEDLQCDIEQVNVKATTTEKLGFT GRQEGIACEAVALLIRQ
1VH8 (A)	4.2	15-32 -> 154-177, C157A	SLIRIGHGFDVHAFVKHASFNITTDVKDRKQKVNATFYFIAHSDGDV ALHALTDAILGAAALGDIGKLFPKNADSRGLLREAFRQVQEKGYKIG NVDITIIAQAPKMRPHIDAMRAKIAEDLQCDIEQVNVKATTTEKLGFT GRQEGIACEAVALLIRQ
1VH8 (A)	4.3	15-32 -> 154-177, C157A, V154G, Y177G	SLIRIGHGFDVHAFGKHASFNITTDVKDRKQKVNATFGFIAHSDGDV ALHALTDAILGAAALGDIGKLFPKNADSRGLLREAFRQVQEKGYKIG NVDITIIAQAPKMRPHIDAMRAKIAEDLQCDIEQVNVKATTTEKLGFT GRQEGIACEAVALLIRQ
1X3E (A)	5.1	35-49 -> GS, delete 111-119, 83-98 -> 154-177)	GDTTITVVGNLTADPELRFTPSGAAVANFTVASTGSALFLRCNIWR EAAENVAESLTRGSRVIVTGRLKVKHCSFNITTDVKDRKQKVNATF YEVEVDEIGPSLR
1X3E (A)	5.2	35-49 -> GS, delete 111-119, 83-98 -> 154-177, C157A)	GDTTITVVGNLTADPELRFTPSGAAVANFTVASTGSALFLRCNIWR EAAENVAESLTRGSRVIVTGRLKVKHASFNITTDVKDRKQKVNATF YEVEVDEIGPSLR
1X3E (A)	5.3	35-49 -> GS, delete 111-119, 83-98 -> 154-177, C157A, K82C + E99C	GDTTITVVGNLTADPELRFTPSGAAVANFTVASTGSALFLRCNIWR EAAENVAESLTRGSRVIVTGRLCVKHASFNITTDVKDRKQKVNATF YCVEVDEIGPSLR

3L1E (A)	-	delete 88-105, 41-55 -> 154-177	SGISEVRSDRDKFVIFLDVKHFSPEDLTVKVQEDFVEIHGVKHCSFN ITTDVKDRKQKVNATFYFHRRYRLPSNVDQSALSCSLSADGMLTFS GPK
3L1E (A)	-	delete 88-105, 41-55->154-177, C157A	SGISEVRSDRDKFVIFLDVKHFSPEDLTVKVQEDFVEIHGVKHASFN ITTDVKDRKQKVNATFYFHRRYRLPSNVDQSALSCSLSADGMLTFS GPK
1DHN (A)	-	100-114 -> 154-177	MQDTIFLKGMRFYGYHGALSAENEIGQIFKVDVTLKVDLSEAGRTD NVIDTVHYGEVFEEVKSIMEGKAVNLLEHLAERIANRINSQYNRVME TKVRIVKHCSFNITTDVKDRKQKVNATFYIEIVRENK
1DHN (A)	-	100-114 -> 154-177, C157A	MQDTIFLKGMRFYGYHGALSAENEIGQIFKVDVTLKVDLSEAGRTD NVIDTVHYGEVFEEVKSIMEGKAVNLLEHLAERIANRINSQYNRVME TKVRIVKHASFNITTDVKDRKQKVNATFYIEIVRENK
1BM9 (A)	-	68-89 -> 154-177	EEKRSSTGFLVKQRAFLKLYMITMTEQERLYGLKLLEVLRSEFKEIG FKPNHTEVYRSLHELLDDGIVKHCSFNITTDVKDRKQKVNATFYKD YEAAKLYKKQLKVELDRCKKLIEKALSDNF
1BM9 (A)	-	68-89 -> 154-177, Y177F, C157A	EEKRSSTGFLVKQRAFLKLYMITMTEQERLYGLKLLEVLRSEFKEIG FKPNHTEVYRSLHELLDDGIVKHASFNITTDVKDRKQKVNATFFKDY EAAKLYKKQLKVELDRCKKLIEKALSDNF
1BM9 (A)	-	68-89 -> 154-177, Y177F, C157A, L33G	EEKRSSTGFLVKQRAFLKLYMITMTEQERLYGGKLLEVLRSEFKEIG FKPNHTEVYRSLHELLDDGIVKHASFNITTDVKDRKQKVNATFYKD YEAAKLYKKQLKVELDRCKKLIEKALSDNF

[a] Listed items include epitope-scaffold name, brief description of epitope transplantation and mutations made in the computational design, and amino acid sequence of the epitope-scaffold.

Table S3. Design and sequence information of 20 glycan V3-supersite transplants.

PDB ID (Chain ID)	Glycan V3-supersite transplant	Epitope transplantation and mutations	Amino-acid sequence
3I8Z (A)	01	R10N + I11C + R12T + insert(miniV3) + R15H + V16C + E17N + L19S	FAVESIEKKNCTRPNNNTRPGEIIGDIRQAHCNYSVKWRGWSPKYNTWEPEENILDPRLLIAFQNR
3HSH (A)	02	V37N + R38C + V39T + insert(miniV3) + G42H + F43C + R44N + V46T	GSSGVRLWATRQAMLGQVHEVPEGWLIFVAEQEELYNCTRPNNNTRPGEIIGDIRQAHCNKTQLEARTPLPR
1RH6 (A)	-	D37T + E40H + L42N + H44T + insert(miniV3)	MYLTLQEWNARQRRPRSLETVRRWVRESRIFPPPVKTRPNNNTRPGEIIGDIRQAHYNFTESAVKVDLNRP
3C4S (A)	-	R26N + V27C + S28T + K32H + A33C + A34N + L36T + insert(miniV3)	MIFPGATVRVTNVDDTYYRFEGLVQNCTRPNNNTRPGEIIGDIRQAHCNVTFENGNWDKLVTFRLSELEAVKP
3LWE (A)	03	K14N + T15C + E16T + K19H + V20C + L21N + k23T + insert(miniV3)	GEDVFEVEKILDMNCTRPNNNTRPGEIIGDIRQAHCNYTVRWKGYTSDDDTWEPEIHLEDCKEVLLEFRKKIAENKA
1MI0 (A)	04	T56H + T58N + insert(miniV3)	MDTYKLVIVLNGTTFTYTTEAVDAATAEKVFKQYANDNGVDGEWTYARPNNNTRPGEIIGDIRQAHFNVTE
2CC6 (A)	-	G42N + V43C + E44T + insert(miniV3) + T52H + Y53C + Q54N + E56S	VFKKVLLTGTSEESFTAAADDAIDRAEDTLDNVVWAEVVDQNCTRPNNNTRPGEIIGDIRQAHCNTSVQVAFELD
1NXB (A)	05	W29N + S30C + D31T + insert(miniV3) + G34H + T35C + I36N + E38S	RICFNQHSSQPQTTKTCSPGESSCYHKQNCTRPNNNTRPGEIIGDIRQAHCNISRGCGCPTVKPGIKLSCCESEVCNN
1EAY (C)	-	V31N + K32C + G33T + insert(miniV3) + S36H + L37C + S38N + I40T	PRRIILSRLKAGEVDLLEEELGHLTTLTDVNCTRPNNNTRPGEIIGDIRQAHCNATLPGDIAEDDITAVLCFVIEADQITFET
2VQC (A)	-	Q58N + Y59C + K60T + insert(miniV3) + K63H + T64C + T65N + K67T	TLNSYKMAEIMYKILEKKGELTLEDILAQFEISVPSAYNIQRALKAICERHPDECEVNCTRPNNNTRPGEIIGDIRQAHCNFTWIK
1I8F (A)	06	E45N + I46C + I47T + insert(miniV3) + N50H + V51C + Y52N + R54S	ATLGATLQDSIGKQVLVKLRDSHEIRGILRSFDQHVNLLLEDAENCTRPNNNTRPGEIIGDIRQAHCNKSGTMVVRGENVLFISPVP
1DUR (A)	07	G26T + I28H + A30N + D33S + insert(miniV3)	AYVINDSCIACGACKPECPVNCIQETRPNNNTRPGEIIGDIRQAHYNISADSCIDCGSCASVCPVGAPNPED
1FR3 (A)	08	V17N + K18C + G19T + insert(miniV3) + M22H + A23C + K24N + V26T	MKISGRNKLEATVKEINCTRPNNNTRPGEIIGDIRQAHCNITMDYKGTELVAAITIDSVADLDLVPGDKVTALVKATEMEVLK
1DP7 (P)	-	L59N + G60C + insert(miniV3) + N64H + S65C + K66N + H68T	TVQWLLDNYETAEGVSLPRSTLYNHYLLHSQEQKLEPVNAASFGKLIRSVFMGLRTRRNCTRPNNNTRPGEIIGDIRQAHCNYTYYGLRIKA
1GUT (A)	09	K16N + K17C + G18T + insert(miniV3) + T21H + A22C + E23N + V25T	SISARNQLKGKVVGLNCTRPNNNTRPGEIIGDIRQAHCNVTLEIAGGNKITSIISLDSVEELGVKEGAELTAVVKSTDVMILA
2AST (C)	-	Y53A + M54A + R66A + Y8N + D9C + D10T + insert(miniV3) + F13H + E14C + Y15N + H17S	QIYYSDKNCTRPNNNTRPGEIIGDIRQAHCNRSVMLPKDIAKLVPKTHLMSESEWRNLGVQQSQGWVHAAIHEPEPHILLFARPL
1WHZ (A)	-	M23N + A24C + K25T + insert(miniV3) + R30C + L31N	MWMPPRPEEVARKLRRLGFVERNCTRPNNNTRPGEIIGDIRQAHCNYTHPDGRIVVVPFHSGELPKGTFKRILRDAGLTEEEFHNL
2P09 (A)	10	K30N + V31C + K32T + insert(miniV3) + L36C + R37N + Y39T	DDDDKKTNWLKRIYRVRPCVKCKVAPRDWNCTRPNNNTRPGEIIGDIRQAHCNITNMCKTCFNNSIDIGDDTYHGHVDWLMYADS
3CA7 (A)	11	F23E + A24I + V25N + K26C + I27T + L30H + P31C + V32N + Y33I + Insert(miniV3) + F2A	TAPTYKCPETFDAWYCLNDAHCEINCTRPNNNTRPGEIIGDIRQAHCNISCECAIGFMGQRCEYKE
3FRY (A)	-	delete1-14 + K36N + V37C + D38T + insert(miniV3) + E41H + A42C + V43N + A45S	SCHHCVARVKKALEEAGAKVENCTRPNNNTRPGEIIGDIRQAHCNVSGNKEDVDKYIKAVEAAGYQAKLRSSAWS

[a] Listed items include epitope-scaffold name, brief description of epitope transplantation and mutations made in the computational design, and amino acid sequence of the epitope-scaffold.

Table S4. Design and sequence information of ferritin nanoparticles of glycan V3-supersite transplants.

Glycan V3-supersite transplant	Amino acid sequence of ferritin nanoparticles*
07	AYVINDSCIACGACKPECPVNCIQETRPNNNTRPGEIIGDIRQAHYNISADSCIDCGSCASVCPVGAPNPED<u>GGGGSG</u>ESQVRQQFSKDIEKLLNEQVNKEMQSSNLYMSMSSWCYTHSLDGAGLFLFDHAAEEYEHAKKLIIFLNENNVPVQLTSISAPEHKFEGLTQIFQKAYEHEQHISESINNIVDHAIKSKDHATFNFLQWYVAEQHEEEVLFKDILDKIELIGNENHGLYLADQYVKGIAKSRKS
08	MKISGRNKLEATVKEINCTRPNNNTRPGEIIGDIRQAHCNITMDYKGTELVAAITIDSVADLDLVPGDKVTALVKATEMEVLK<u>GGGGSG</u>ESQVRQQFSKDIEKLLNEQVNKEMQSSNLYMSMSSWCYTHSLDGAGLFLFDHAAEEYEHAKKLIIFLNENNVPVQLTSISAPEHKFEGLTQIFQKAYEHEQHISESINNIVDHAIKSKDHATFNFLQWYVAEQHEEEVLFKDILDKIELIGNENHGLYLADQYVKGIAKSRKS
09	SISARNQLKGKVVGLNCTRPNNNTRPGEIIGDIRQAHCNVTLEIAGGNKITSIISLDSVEELGVKEGAELTAVVKSTDVMILA<u>GGGGSG</u>ESQVRQQFSKDIEKLLNEQVNKEMQSSNLYMSMSSWCYTHSLDGAGLFLFDHAAEEYEHAKKLIIFLNENNVPVQLTSISAPEHKFEGLTQIFQKAYEHEQHISESINNIVDHAIKSKDHATFNFLQWYVAEQHEEEVLFKDILDKIELIGNENHGLYLADQYVKGIAKSRKS
10	DDDDKKTNWLKRIYRVRPCVKCKVAPRDWNCTRPNNNTRPGEIIGDIRQAHCNITNMCKTCFNNSIDIGDDTYHGHVDWLMYADS<u>GGGGSG</u>ESQVRQQFSKDIEKLLNEQVNKEMQSSNLYMSMSSWCYTHSLDGAGLFLFDHAAEEYEHAKKLIIFLNENNVPVQLTSISAPEHKFEGLTQIFQKAYEHEQHISESINNIVDHAIKSKDHATFNFLQWYVAEQHEEEVLFKDILDKIELIGNENHGLYLADQYVKGIAKSRKS
11	TAPTYKCPETFDAWYCLNDAHCEINCTRPNNNTRPGEIIGDIRQAHCNISCECAIGFMGQRCEYKE<u>GGGGSG</u>ESQVRQQFSKDIEKLLNEQVNKEMQSSNLYMSMSSWCYTHSLDGAGLFLFDHAAEEYEHAKKLIIFLNENNVPVQLTSISAPEHKFEGLTQIFQKAYEHEQHISESINNIVDHAIKSKDHATFNFLQWYVAEQHEEEVLFKDILDKIELIGNENHGLYLADQYVKGIAKSRKS

* Linker sequences between glycan V3-supersite transplants and ferritin are underlined.
